# Electrophysiological and calcium-handling development during long-term culture of human-induced pluripotent stem cell-derived cardiomyocytes

**DOI:** 10.1007/s00395-022-00973-0

**Published:** 2023-04-05

**Authors:** Fitzwilliam Seibertz, Henry Sutanto, Rebekka Dülk, Julius Ryan D. Pronto, Robin Springer, Markus Rapedius, Aiste Liutkute, Melanie Ritter, Philipp Jung, Lea Stelzer, Luisa M. Hüsgen, Marie Klopp, Tony Rubio, Funsho E. Fakuade, Fleur E. Mason, Nico Hartmann, Steffen Pabel, Katrin Streckfuss-Bömeke, Lukas Cyganek, Samuel Sossalla, Jordi Heijman, Niels Voigt

**Affiliations:** 1 Institute of Pharmacology and Toxicology, University Medical Center Göttingen, Georg-August University Göttingen, Universitätsmedizin Göttingen, Robert-Koch-Straße 40, 37075 Göttingen, Germany; 2 DZHK (German Center for Cardiovascular Research), Partner Site Göttingen, Göttingen, Germany; 3 Cluster of Excellence “Multiscale Bioimaging: From Molecular Machines to Networks of Excitable Cells” (MBExC), University of Göttingen, Göttingen, Germany; 4 Department of Cardiology, Cardiovascular Research Institute Maastricht, Faculty of Health, Medicine and Life Sciences, Maastricht University, Universiteitssingel 50, 6229 ER Maastricht, The Netherlands; 5 Nanion Technologies GmbH, Munich, Germany; 6 Clinic for Cardiology and Pneumology, University Medical Center Göttingen, Georg-August University Göttingen, Göttingen, Germany; 7 Department of Internal Medicine II, University Medical Center Regensburg, Regensburg, Germany; 8 Institute of Pharmacology and Toxicology, University of Würzburg, Würzburg, Germany

**Keywords:** Stem cell, Calcium handling, Maturation, Ion channel, Action potential, Cardiovascular

## Abstract

Human-induced pluripotent stem cell-derived cardiomyocytes (hiPSC-CMs) are increasingly used for personalised medicine and preclinical cardiotoxicity testing. Reports on hiPSC-CM commonly describe heterogenous functional readouts and underdeveloped or immature phenotypical properties. Cost-effective, fully defined monolayer culture is approaching mainstream adoption; however, the optimal age at which to utilise hiPSC-CM is unknown. In this study, we identify, track and model the dynamic developmental behaviour of key ionic currents and Ca^2+^-handling properties in hiPSC-CM over long-term culture (30–80 days). hiPSC-CMs > 50 days post differentiation show significantly larger *I*_Ca,L_ density along with an increased *I*_Ca,L_-triggered Ca^2+^-transient. *I*_Na_ and *I*_*K*1_ densities significantly increase in late-stage cells, contributing to increased upstroke velocity and reduced action potential duration, respectively. Importantly, our in silico model of hiPSC-CM electrophysiological age dependence confirmed *I*_*K*1_ as the key ionic determinant of action potential shortening in older cells. We have made this model available through an open source software interface that easily allows users to simulate hiPSC-CM electrophysiology and Ca^2+^-handling and select the appropriate age range for their parameter of interest. This tool, together with the insights from our comprehensive experimental characterisation, could be useful in future optimisation of the culture-to-characterisation pipeline in the field of hiPSC-CM research.

**Supplementary Information:**

The online version contains supplementary material available at 10.1007/s00395-022-00973-0.

## Introduction

Human-induced pluripotent stem cell-derived cardiomyocytes (hiPSC-CMs) show immense promise for the cost-effective development of personalised medicine and the streamlining of preclinical cardiotoxicity testing [1]. Derived from blood or minimally invasive patient biopsies, in vitro hiPSC-CM constructs preserve patient-specific genotypes, are highly scalable and avoid the practical and ethical pitfalls associated with primary human tissue culture and animal experimentation [79]. hiPSC-CM technology has contributed to the emergence of initiatives such as the comprehensive in vitro proarrhythmia assay (CiPA) approach where multimodal examinations of drug responses aim to provide a more robust assessment of proarrhythmic risk [17, 60].

At present, in vitro hiPSC-CM technology is limited both by a persistent state of phenotypic immaturity and highly heterogenous readouts of electrophysiological function. The latter could simply reflect the genetic variability inherent in the general population; however, even hiPSC-CM derived from the same donor and within the same cell-line can demonstrate large phenotypic variability [13]. Variability could arise from numerous sources including differentiation methods, plating densities, or indeed the age at which the hiPSC-CM construct is assayed. hiPSC-CM morphology and function can evolve over long culture periods [49]. Functional expression of major ionic currents including the transient-outward K^+^ current (*I*_to_) and L-type Ca^2+^ current (*I*_Ca,L_) increases in human embryonic stem cell-derived cardiomyocytes (hESC-CM) cultured for several weeks [64], recapitulating electrophysiological embryonic development described in animal models [30]. In hiPSC-CM, action potential (AP) characteristics have also been reported to change haphazardly during long culture periods, prompting the notion of temporal fluidity in the dominant cardiac subtype within in vitro cultures [3]. These findings suggest the presence of complex non-linear changes in ion channel characteristics throughout cell culture. Although cardiomyocyte Ca^2+^-handling is essential for excitation–contraction coupling and plays a major role in arrhythmogenesis [67], a comparative readout of Ca^2+^-handling has yet to be reported in hiPSC-CM over 30 days post differentiation [11, 27, 48]. There is little standardisation of the ages at which hiPSC-CMs are employed for drug screening or modelling purposes [3, 11]. We hypothesise that developmental processes during long-term hiPSC-CM culture may contribute to the phenotypic variability frequently reported within and between laboratories. Therefore, the present work characterises the passive maturation of hiPSC-CM electrophysiology and Ca^2+^-handling during long-term culture. Finally, to evaluate whether our experimentally observed age-dependent changes in Ca^2+^-handling parameters and major ionic currents are sufficient to explain the experimentally acquired AP characteristics, we have integrated our experimental data into an in silico framework based on recent hiPSC-CM-specific in silico models of cardiac cellular electrophysiology [36, 40, 54, 55].

## Methods

Further details of all methods can be found in the Online Data Supplement.

### Somatic cell reprogramming and cardiac differentiation

hiPSC cell line UMGi014-C clone 14 (isWT1.14) was derived from the dermal fibroblasts of a healthy male donor (31 years). They were cultured in feeder-free conditions using the integration-free CytoTune iPS 2.0 Sendai Reprogramming Kit (Thermo Fisher Scientific) with reprogramming factors OCT4, KLF4, SOX2, c-MYC. Previously published pluripotency and karyotype analysis of this line revealed no abnormalities or chromosomal instability [59]. Experimental protocols were approved by the ethics committee of the University Medical Center Göttingen (10/9/15). Directed feeder-free cardiac differentiation was achieved via canonical WNT modulation with small-molecules CHIR and IWP2, followed by metabolic selection with lactate as previously described [19, 39]. Day-3 (d-3) indicates final passaging whilst day 0 (d0) marks the onset of differentiation with WNT stimulation.

### Cellular preparation

**Fig. 1:**
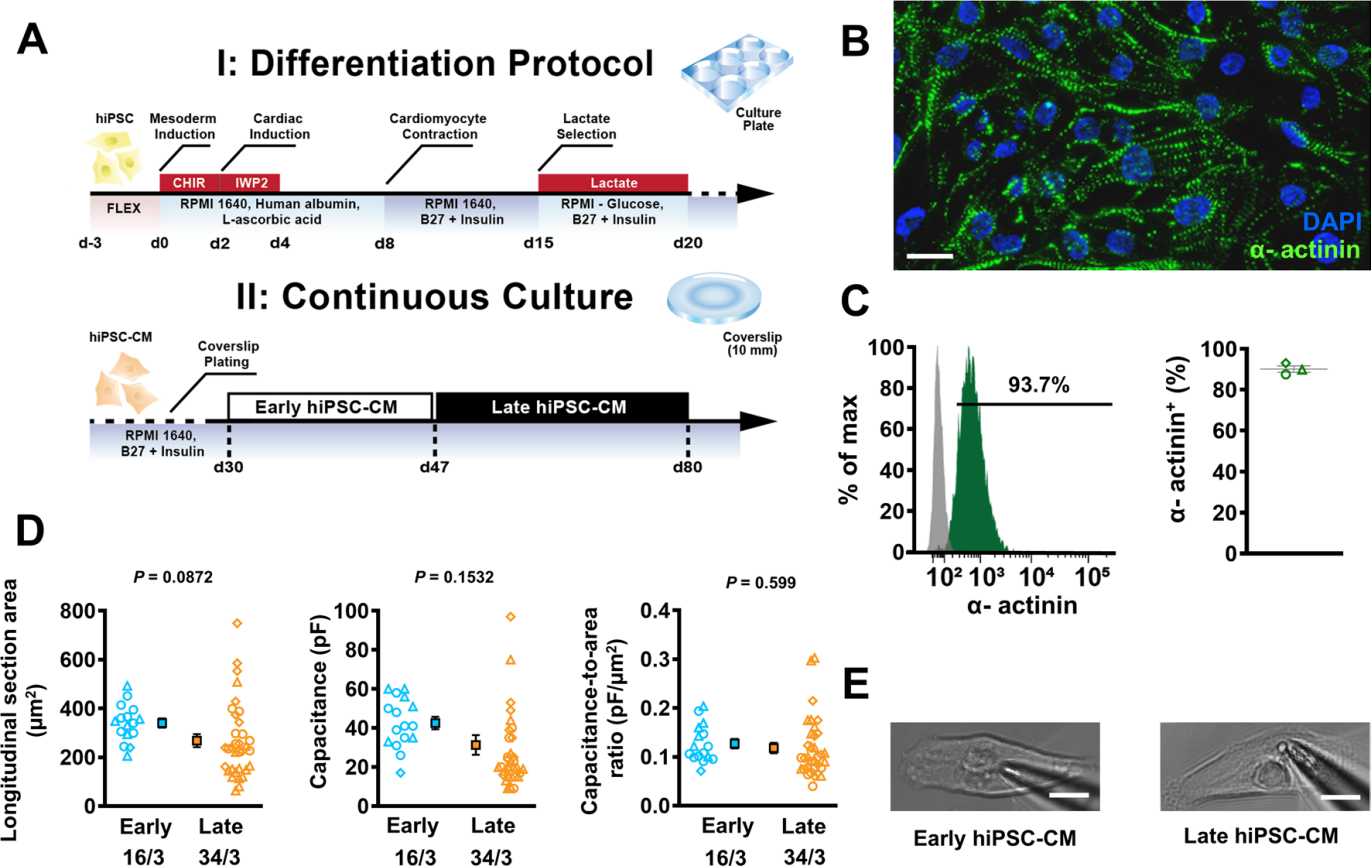
Overview of human induced pluripotent stem cell-derived cardiomyocytes (hiPSC-CM) differentiation. **A** Schematic overview of the differentiation protocol utilised in this study (upper), and the process of long-term continuous culture on glass coverslips (lower). Early (young) hiPSC-CM underwent experimentation between 30 and 46 days after differentiation whilst late (old) hiPSC-CM were measured between day 47 to 80. **B** Immunofluorescent staining of hiPSC-CM at d29. **C** Flow cytometry analysis of hiPSC-CM at d29. **D** Longitudinal section area of early and late hiPSC-CM (left), corresponding cell capacitance (middle) and T-tubule density (right), estimated through a ratio of capacitance to longitudinal section area of each cell.** E** Representative photomicrographs of early (left) and late (right) hiPSC-CM. Scale bar represents 10 µm. Data are mean ± SEM. Symbols represent separate differentiations. *n*/*N* = number of hiPSC-CM/differentiation

Between d27 and d30, purified hiPSC-CMs were digested with TrypLE (Thermo Fisher Scientific) and sparsely plated on 1:60 Matrigel-coated borosilicate glass 10 mm #0 round coverslips at a density of 15,000 cells/cm^2^. Cells were incubated at 37 °C in 5% CO_2_ and maintained every 2–3 days with a culture medium containing RPMI 1640 supplemented with B-27 (both Thermo Fisher Scientific). Cellular beating rate was routinely measured by photometric capture at × 40 magnification with a Retiga R6™ CCD camera mounted on an inverted microscope. Recordings were taken at 13 frames per second and analysed offline using the MUSCLEMOTION™ plugin on ImageJ [62]. For experimentation, coverslips were removed from their media and inserted directly into a heated bath chamber mounted on the stage of an inverted epifluorescence microscope. The differentiation and preparation process is outlined in Fig. 1a.

### Electrophysiological recordings

Whole-cell ruptured-patch techniques were employed to measure membrane currents in single, isolated early (d30–d46) and late (d47–d80) stage hiPSC-CMs. *I*_Ca,L_ and intracellular Ca^2+^ were measured simultaneously at 0.5 Hz with a voltage-clamp protocol consisting of a 100-ms ramp to − 40 mV (inactivating the fast Na^+^ current; *I*_Na_) followed by a 100-ms depolarising test-pulse to + 10 mV, as previously described [15, 77]. For current voltage (I–V) curves, the test pulse was altered from − 40 to + 60 mV with 5 mV steps. Bath solution contained (in mmol/L): CaCl_2_ 2, glucose 10, HEPES 10, KCl 4, MgCl_2_ 1, NaCl 140, probenecid 2; pH = 7.35. 4-aminopyridine (5 mmol/L) and BaCl_2_ (0.1 mmol/L) were added to block K^+^ currents [8, 73, 74, 78]. Pipette solution contained (in mmol/L): Fluo-3 pentapotassium salt 0.1, EGTA 0.02, GTP-Tris 0.1, HEPES 10, K-aspartate 92, KCl 48, Mg-ATP 1, Na_2_-ATP 4; pH = 7.2. Sarcoplasmic reticulum (SR) Ca^2+^ content was assessed through integration of the Na^+^/Ca^2+^ exchanger-mediated current (*I*_NCX_) during perfusion with 10 mmol/L caffeine.

Peak *I*_Na_ was measured in a bath solution containing (in mmol/L): NaCl 5, HEPES 10, MgCl_2_ 1, CsCl 10, glucose 10, CaCl_2_ 0.5, and TEA-Cl 120 (pH = 7.4, adjusted with CsOH). A voltage-clamp protocol was applied consisting of a holding potential at − 80 mV, followed by a 1000-ms pre-pulse step at − 110 mV (to increase availability of Na^+^ channels), and then 30-ms steps from − 80 to + 20 mV for I–V curves. Late Na^+^ current (*I*_Na,L_) measurements were conducted with a bath solution containing (in mmol/L): NaCl 120, HEPES 10, MgCl_2_ 1, CsCl 10, glucose 10 and CaCl_2_ 0.5 (pH = 7.4, adjusted with CsOH). A voltage-clamp protocol was applied consisting of a holding potential of − 120 mV, followed by a 5-ms activating step to + 50 mV and a 300-ms step to − 30 mV to assess *I*_Na,L_. Pipette solution for both *I*_Na_ and *I*_Na,L_ measurements contained (in mmol/L): NaCl 5, EGTA 10, GTP-Tris 0.4, HEPES 10, Mg-ATP 4, CsCl 20, CaCl_2_ 3, Cs-Methansulfonate 90, pH = 7.2.

Delayed rectifier (rapid component; *I*_Kr_) tail currents were measured using a high-performance automated patch clamp system (SyncroPatch 384; Nanion Technologies GmbH) with a voltage-clamp protocol consisting of a holding potential of − 80 mV followed by a 2-s step to 60 mV with steps of 10 mV for I–V acquisition. Bath solution contained (in mmol/L): CsCl 144, CaCl_2_ 2, MgCl_2_ 2, glucose 5, HEPES 10 (pH = 7.4 adjusted with CsOH). Pipette solution contained (in mmol/L) CsCl 20, EGTA 10, HEPES 10, CsF 110 (pH = 7.2 adjusted with CsOH) in accordance with a recently published protocol [26]. Cs^+^ was used as a charge carrier due to its selectivity for the hERG channel [65].

Basal inward-rectifier K^+^ current (*I*_*K*1_) was measured at 0.5 Hz with a ramp pulse from − 100 to + 40 mV at 0.5 Hz while superfusing a modified Tyrode’s bath solution containing (mmol/L): NaCl 120, KCl 20, MgCl_2_ 1, CaCl_2_ 2, glucose 10, HEPES 10, pH = 7.4. Pipette solution contained (in mmol/L): EGTA 0.02, GTP-Tris 0.1, HEPES 10, K-aspartate 92, KCl 48, Mg-ATP 1, Na_2_-ATP 4; pH = 7.2. *I*_*K*1_ was identified as Ba^2+^ (1 mmol/L)-sensitive current as previously described [76].

APs were measured in current-clamp configuration at 0.5 Hz in bath solution containing (in mmol/L) the following: CaCl_2_ 2, glucose 10, HEPES 10, KCl 4, MgCl_2_ 1, NaCl 140; pH = 7.35. Pipette solution contained (in mmol/L): EGTA 0.02, GTP-Tris 0.1, HEPES 10, K-aspartate 92, KCl 48, Mg-ATP 1, Na_2_-ATP 4; pH = 7.2. Mean holding currents were − 0.86 ± 0.13 pA/pF for early hiPSC-CM and − 1.05 ± 0.16 pA/pF for late hiPSC-CM (*P* = 0.3651).

All electrophysiological experiments were carried out at 37 °C, except for *I*_Na_, *I*_Na,L_ and *I*_Kr_ which were measured at room temperature. Seal resistances were 3–6 GΩ. Borosilicate glass pipettes with tip resistances of 2–7 MΩ were used for voltage clamp experiments. High resistance borosilicate glass pipettes (5–10 MΩ) were used for current clamp experiments. All current recordings (except for *I*_Kr_) were acquired using an Axopatch 200B microelectrode amplifier and analysed using pClamp-Software V 10.7 (both from Axon Instruments Inc., Foster City, USA). Membrane currents were corrected for membrane capacitance and expressed in pA/pF. Action potentials were acquired using a HEKA amplifier and HEKA patchmaster software and analysed using Lab Chart 7 (AD instruments, Otago, New Zealand).

### Simultaneous intracellular Ca^2+^ measurements

[Ca^2+^]_i_ of single, isolated early- and late-stage hiPSC-CM was measured using the fluorescent Ca^2+^ indicator fluo-3-acetoxymethyl ester (Fluo-3-AM, 10 µmol/L, 10 min loading, 30 min de-esterification, *λ*_Ex_ = 488 nm, *λ*_Em_ = 535 nm) during simultaneous *I*_Ca,L_ measurement at 37 °C as previously described [77]. Fluorescence emission was collected with a photomultiplier optimised for high-speed signal capture (10 kHz). Emission was correlated to [Ca^2+^]_i_ with the formula [Ca^2+^]_i_ = *K*_d_ [*F*/(*F*_max_ − *F*)]. Here, *K*_d_ represents the dissociation constant of Fluo-3 (864 nmol/L), F denotes Fluo-3 fluorescence, and *F*_max_ describes Ca^2+^-saturated fluorescence obtained through cellular laceration at the end of each experiment [15].

### Molecular biology studies

Early and late hiPSC-CM were trypsinised and cellular membranes were isolated by differential centrifugation and then solubilized at 1 mg/ml of total protein in solubilisation buffer. Ca^2+^-handling proteins SERCA2a and NCX1, as well as expression of Kir2.1 were analysed with immunoblotting techniques. (LI-COR Biotechnology, US). Antibodies are outlined in Online Table S1. Immunofluorescent screening stained for nuclear and sarcomeric proteins using hiPSC-CM (d29) fixed in 4% PFA and permeabilised in 0.1% Triton X-100 with an AxioObserver A1 fluorescence microscope (Carl Zeiss, Jena, Germany). Flow cytometry utilised trypsinised, fixed and permeabilised hiPSC-CM (d29). Cells were screened using the LSRII flow cytometer (BD Biosciences, US).

### Computational modelling

The state-of-the-art in silico hiPSC-CM model by Kernik et al*.* [36] formed the basis for our simulations. The model was implemented in Myokit [9] and model parameters were adjusted to reproduce experimental data from early- or late-stage hiPSC-CM obtained in the present study (Online Table S2). The model age was set to 30 days for early-stage hiPSC-CM and 50 days for late-stage hiPSC-CM. For optimisation of *I*_*K*1_, 40 days and 60 days were used, based on the maturity levels of the experimental data. Finally, we interpolated the parameters obtained for the early- and late-stage hiPSC-CM models to obtain parameter values as a function of age (Online Table S2, right columns). Linear functions were used for interpolation whenever possible. Alternatively, Hill functions were employed to prevent unphysiological values (e.g., negative membrane capacitance) at advanced age. AP simulations were performed and the steady-state AP following 1000 beats of prepacing was used for analysis in the presence of 0.2 pA/pF hyperpolarising current injected to suppress hiPSC-CM automaticity. The model code is freely accessible at www.github.com/jordiheijman. The installation guide for the induced pluripotent stem cell-derived maturity evaluator (iMATURE) is available in the Online Supplement.

### Statistical analysis

Data are reported as mean ± SEM and *n*-numbers as *n*/*N*, where n indicates number of hiPSC-CM studied from N differentiations, unless otherwise stated. Analyses were carried out with Prism 8 software (Graphpad, San Diego, USA). Normality of the data distribution was assessed using the Shapiro–Wilk normality test. Normally distributed data were compared using unpaired two-tailed Student’s *t*-test unless otherwise indicated. Data with unequal variance were compared using Welch’s *t*-test. Non-normally distributed data were compared using the Mann–Whitney *U* test. *P* < 0.05 was taken as statistically significant.

## Results

In order to generate highly controllable and standardised data sets, we applied a fully defined feeder-free monolayer-based differentiation protocol to our hiPSC cultures (Fig. 1a), directing cardiac induction as previously described [39]. Spontaneous beating was regularly observed by day 8 (d8). Following differentiation, hiPSC-CM stained positive for cardiac marker alpha-actinin, with clear sarcomeric structures visible (Fig. 1b). 90.1 ± 1.6% of cells were alpha-actinin positive, indicating satisfactory differentiation efficiency (Fig. 1c). Following differentiation, hiPSC-CM were plated at low density (15,000/cm^−2^) to ensure regular analysis of single, isolated cells which remain unaffected by electrical and paracrine influences of dense syncytial layers or cellular aggregates. Based on experimental and modelling data, cells assayed between d30 and d46 were designated as early-stage hiPSC-CM while d47–d80 were allocated to the late-stage development category. Cellular size was ascertained through longitudinal cross-sectional area measurement by tracing the perimeter of the hiPSC-CM using the freehand selection tool on ImageJ in a cohort of cells also utilised for patch-clamp experiments. Longitudinal cross-sectional area appeared unchanged in accordance with comparable membrane capacitance. The ratio between cellular area and capacitance indicated limited structural alterations in t-tubule density between early and late cultures (Fig. 1d). Isolated cells with no contact to neighbouring cells and clear membrane resolution were used for electrophysiological characterisation (Fig. 1e).

### Increased systolic Ca^2+^ release from the SR of late hiPSC-CM cultures

**Fig. 2:**
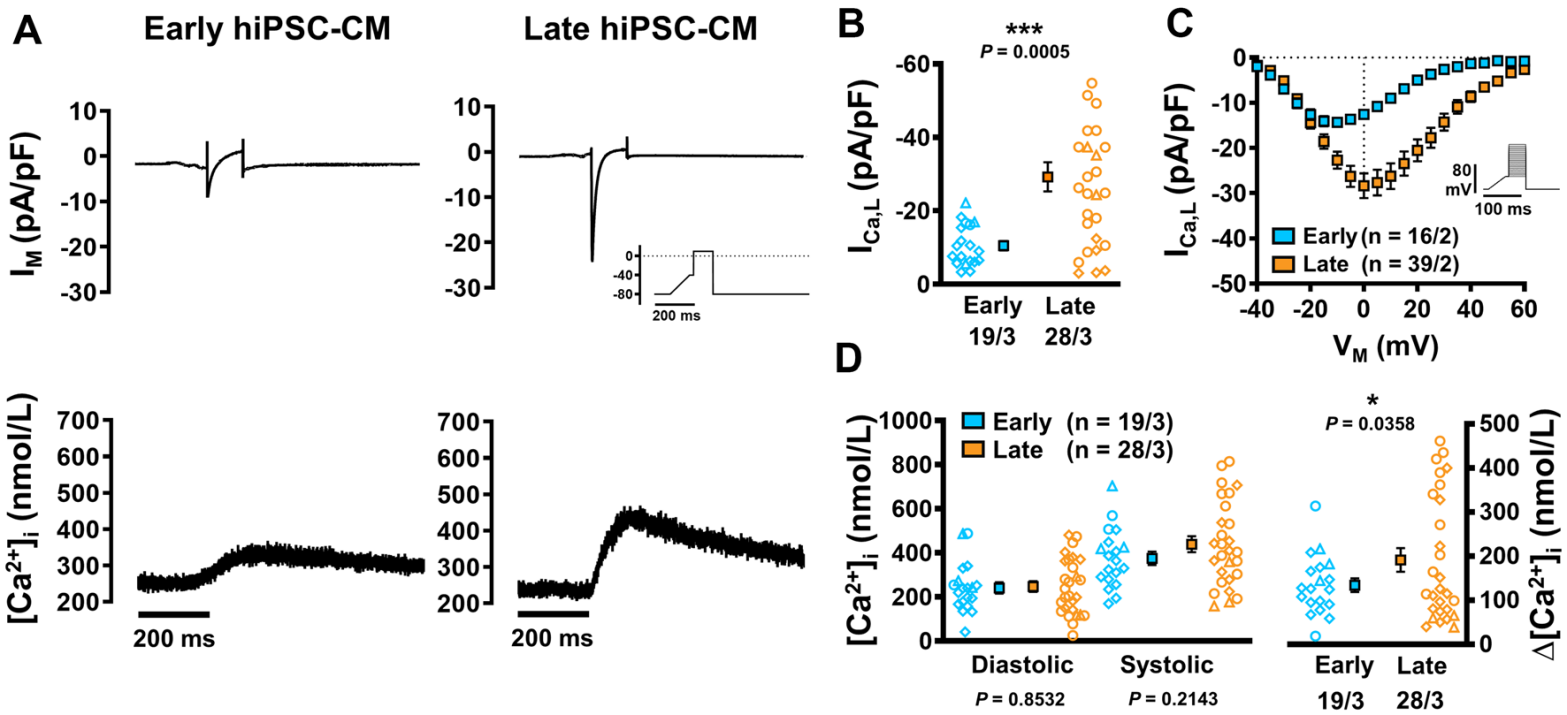
*I*_Ca,L_-triggered Ca^2+^ transients (CaT) in isolated early and late human induced pluripotent stem cell-derived cardiomyocytes (hiPSC-CM).** A** Representative simultaneous recordings of *I*_Ca,L_ (upper) and triggered CaT (lower) in early (left) and late hiPSC-CM (right). Inset: voltage-clamp protocol. **B** Peak *I*_Ca,L_. **C** Current–voltage relationship curve for *I*_Ca,L_. **D** Diastolic and systolic [Ca^2+^]_i_ (left) and resulting CaT-amplitude (right). Data are mean ± SEM. *P < 0.05 ***P < 0.001 versus early hiPSC-CM culture by Welch’s t test or Student’s t test (D left). Symbols represent separate differentiations. *n*/*N* = number of hiPSC-CM/differentiation

Next, we assessed *I*_Ca,L_-triggered Ca^2+^ transients (CaTs) at different stages of hiPSC-CM development. *I*_Ca__,__L_ was initiated by a voltage-clamp protocol and was measured simultaneously with CaT (Fluo-3) in hiPSC-CM (Fig. 2a). Peak *I*_Ca,L_ was significantly larger in late hiPSC-CM versus early culture (− 29.24 ± 3.98 vs. − 10.52 ± 1.27 pA/pF, *n*/*N* = 28/3 vs. 19/3, *P* = 0.0005; Fig. 2b). Current–voltage relationships showed a positive shift in maximal current density in late cultures (Fig. 2c). Diastolic [Ca^2+^]_i_ was similar between both groups; however, systolic [Ca^2+^]_i_ was higher in late cultures. This was matched by a significant increase in CaT amplitude in late cells versus early cells (191.6 ± 26.81 vs. 134.3 ± 15.49 nmol/L, *n*/*N* = 28/3 vs. 19/3, *P* = 0.0358; Fig. 2d). The Ca_v_1.2 blocker nifedipine (1 µmol/L) produced a reduction in *I*_Ca__,__L_ in every instance, suggesting presence of functional Ca_V_1.2 from an early stage in cellular differentiation (Online Fig. S1).

**Fig. 3:**
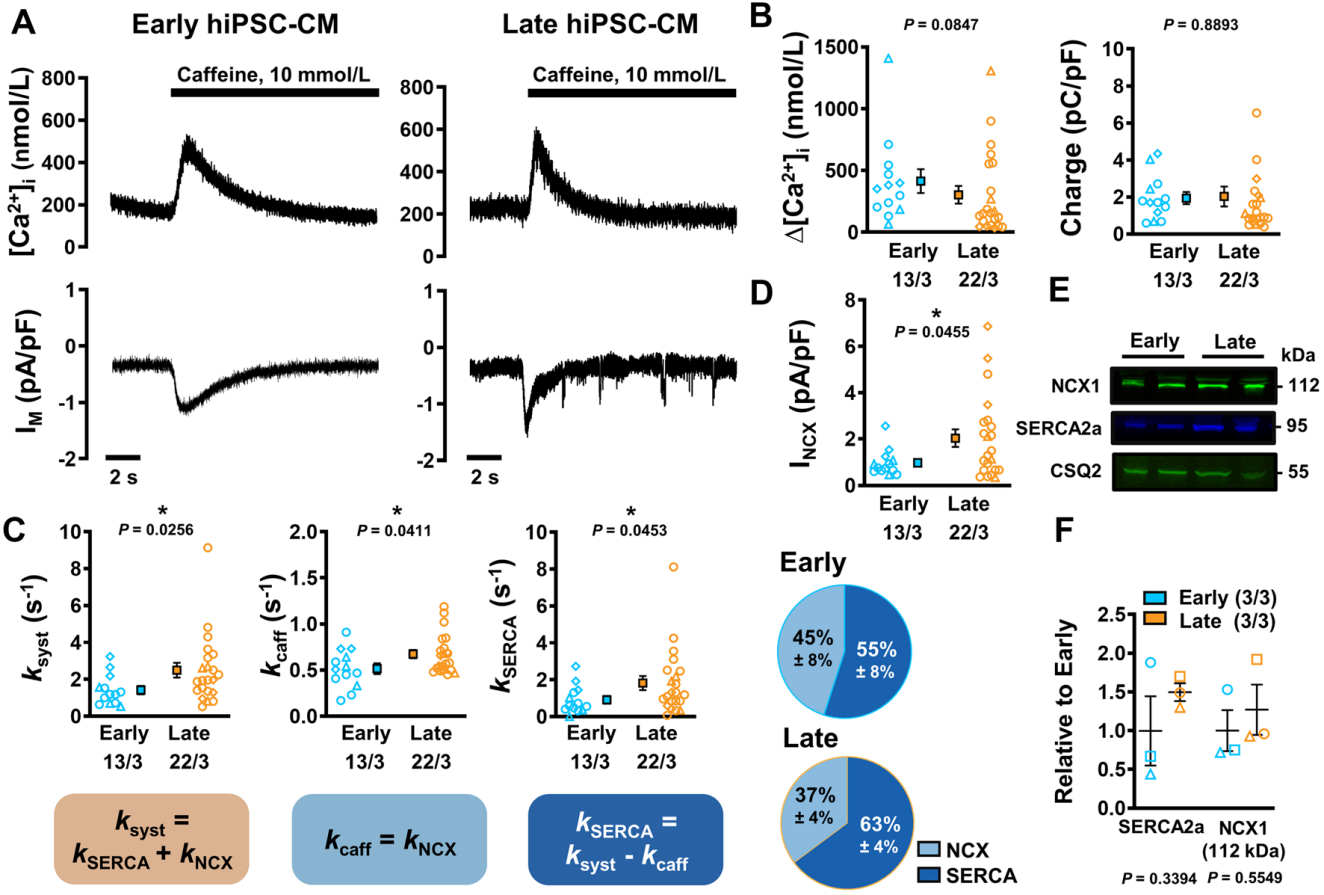
Caffeine-induced Ca^2+^ transients (cCaT) with corresponding transient-inward currents (*I*_NCX_) to assess sarcoplasmic reticulum (SR) Ca^2+^ content in isolated early and late human induced pluripotent stem cell-derived cardiomyocytes (hiPSC-CM). **A** Representative cCaT (upper) and corresponding *I*_NCX_ (lower) in early (left) and late hiPSC-CM (right). **B** SR Ca^2+^ load, quantified as cCaT amplitude (left), or integrated membrane current (Charge; right). **C** Rate constants of Ca^2+^ transport *k*_syst_ (far left), *k*_caff_ (centre left), *k*_SERCA_ (calculated as the difference between *k*_syst_ and *k*_caff_; centre right) and the resulting relative proportions of NCX and SERCA-mediated cytosolic Ca^2+^ removal in early and late hiPSC-CM (far right). **D** Peak *I*_NCX_. **E** Representative western blots showing the expression of NCX1 and SERCA2a against CSQ2. **F** Quantification of NCX1 and SERCA2a expression relative to early hiPSC-CM (3 independent experiments per group). Data are mean ± SEM. *P < 0.05 versus early hiPSC-CM culture by Welch’s t test or Mann–Whitney *U* test (B). Symbols represent separate differentiations. *n*/*N* = number of hiPSC-CM/differentiation

SR Ca^2+^ content was assessed through caffeine application (10 mmol/L) after cessation of the *I*_Ca,L_-activating protocol (Fig. 3a). The resulting caffeine-induced CaT (cCaT) amplitude and integral of corresponding membrane current (reflecting NCX-mediated Ca^2+^ extrusion; charge) were comparable between late and early hiPSC-CM cultures, indicating that higher CaT amplitude in late cells is mainly due to increased trigger *I*_Ca,L_ (Fig. 3b).

### Altered diastolic Ca^2+^-handling in late hiPSC-CM cultures

Diastolic Ca^2+^ removal from the cytosol was faster in late cultures versus early cultures as indicated by the rate constant of systolic CaT decay (inverse of CaT τ, *k*_*syst*_, Fig. 3c). Decay of cCaT mainly reflects NCX-mediated Ca^2+^ removal and was also faster in more mature cells. This is consistent with higher peak *I*_NCX_ density (2.03 ± 0.38 vs. 0.97 ± 0.16 pA/pF, *n*/*N* = 22/3 vs. 13/3, *P* = 0.0455; Fig. 3d), pointing to increased NCX activity in late hiPSC-CM cultures. In accordance, average protein expression of NCX1 was numerically larger in late hiPSC-CM (Fig. 3e, f). Absolute levels of housekeeping protein calsequestrin (CSQ2) showed no difference between early and late cultures (1.00 ± 0.33 vs. 1.03 ± 0.28 a.u relative to early, *n*/*N* = 3/3 vs. 3/3 [not shown]). The rate constant *k*_SERCA_ represents the difference between the rate constant of cCaT decay and that of systolic CaT decay [15]. *k*_SERCA_ was significantly larger in late cultures versus early cultures (1.82 ± 0.38 vs. 0.91 ± 0.21 s^−1^, *n*/*N* = 22/3 vs. 13/3, *P* = 0.0453; Fig. 3c). This was supplemented by the western blot findings (Fig. 3e, f).

Confocal line-scan analysis of Ca^2+^ sparks revealed a tendency towards decreased Ca^2+^ spark frequency and significantly decreased Ca^2+^ leak from the SR in late hiPSC-CM cultures versus early (12.73 ± 5.06 vs. 30.35 ± 9.04 100 µm^−1^ s^−1^, *n*/*N* = 41/7 vs. 39/2, *P* = 0.0416; Online Fig. S2). In addition, spontaneous beating rate, a marker of automaticity, was decreased in late-stage hiPSC-CM (0.45 ± 0.18 vs. 0.79 ± 0.4 Hz, *n*/*N* = 11/2 vs. 12/2, *P* = 0.0067; Online Fig. S2).

### Maturation dependent increase of peak ***I***_Na_ during long-term culture

**Fig. 4:**
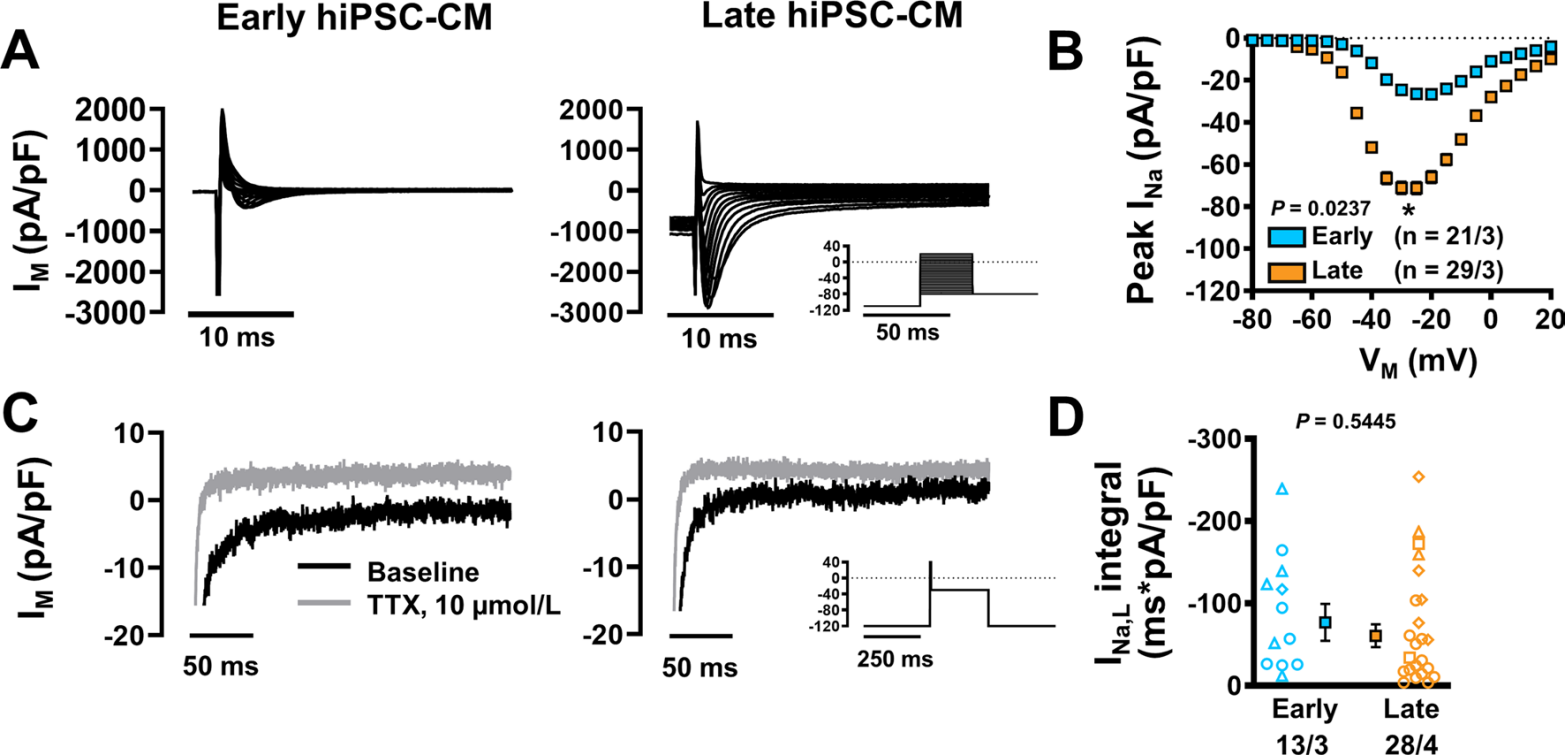
Peak Na^+^ current (*I*_Na_) and late Na^+^ current (*I*_Na,L_) in isolated early and late human induced pluripotent stem cell-derived cardiomyocytes (hiPSC-CM). **A** Representative *I*_Na_ in early (left) and late hiPSC-CM (right). Inset: voltage-clamp protocol. **B** Current–voltage relationship for *I*_Na_. **C** Representative *I*_Na,L_ in early (left) and late hiPSC-CM (right) in the absence (Baseline) or presence of 10 µmol/L tetrodotoxin (TTX). Inset: modified voltage-clamp protocol to accentuate late current (as described in Poulet et al*.* [56]). **D** *I*_Na,L_ integral. Data are mean ± SEM. *P < 0.05 versus early hiPSC-CM culture. Symbols represent separate differentiations. *n*/*N* = number of hiPSC-CM/differentiation

Peak *I*_Na_ was significantly larger in late-stage hiPSC-CM (− 71.12 ± 15.77 vs. − 26.63 ± 4.89 pA/pF, *n*/*N* = 29/3 vs. 21/3; *P* = 0.0237) with current–voltage relationships showing a slight negative shift in peak current density (Fig. 4a, b). *I*_Na,L_ was subsequently measured as current responsive to tetrodotoxin (TTX, 10 µmol/L) in both late and early hiPSC-CM cultures (Fig. 4c). In contrast to peak *I*_Na_, integrated *I*_Na,L_ was not different between early and late hiPSC-CM (Fig. 4d).

### Emergence of robust ***I***_***K***1_ during long-term culture

**Fig. 5:**
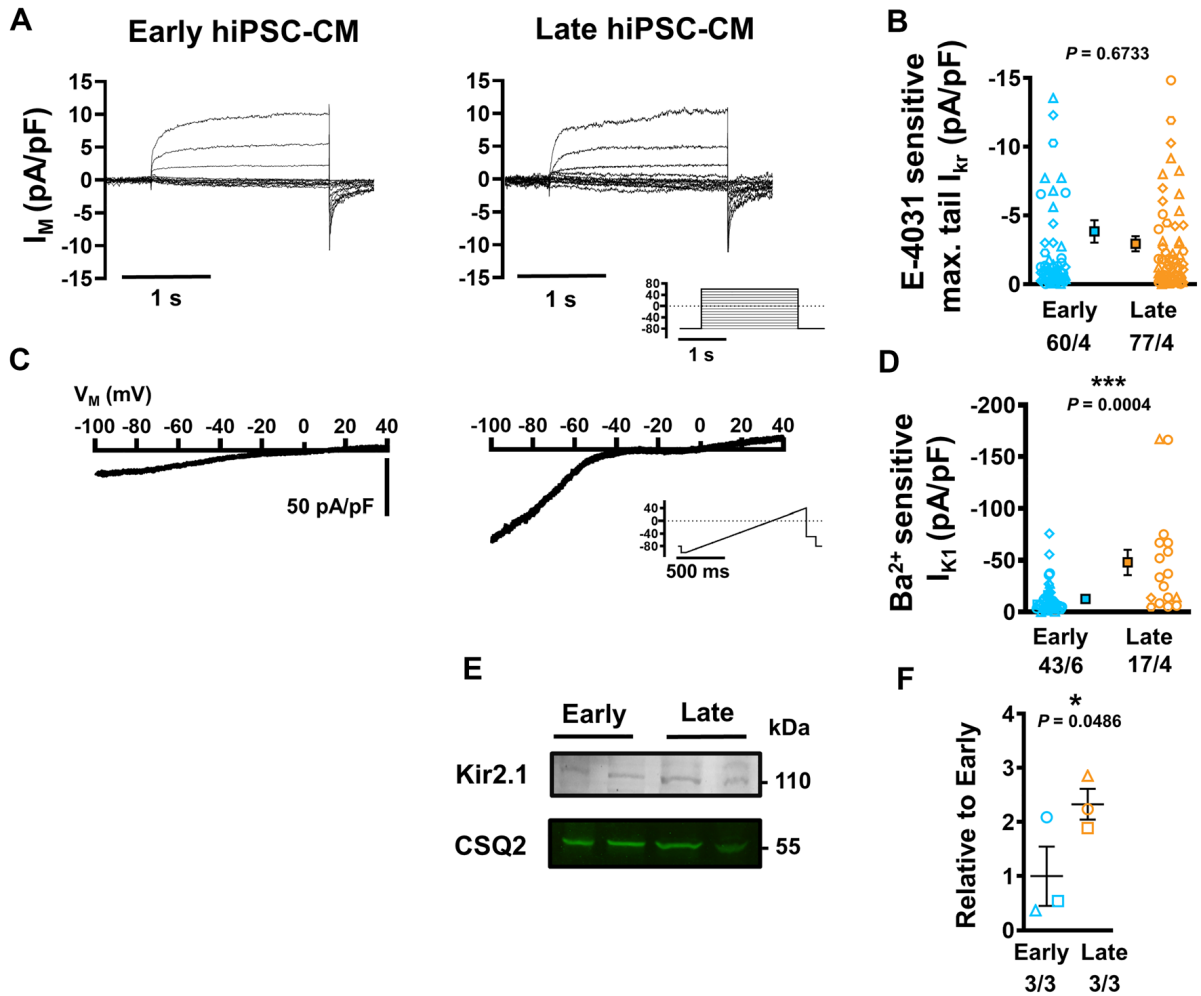
Rapid component of the delayed-rectifier K^+^ current (*I*_Kr_) and basal inward-rectifier K^+^ current (*I*_*K*1_) in isolated early and late human induced pluripotent stem cell-derived cardiomyocytes (hiPSC-CM). **A** Representative *I*_Kr_ in early (left) and late hiPSC-CM (right). Inset: voltage-clamp protocol. **B** Maximum tail *I*_Kr_ defined as E4031-sensitive current. **C** Representative recordings of *I*_*K*1_ in early (left) and late hiPSC-CM (right) during a depolarising ramp pulse protocol (inset). **D** Peak *I*_*K*1_ defined as Ba^2+^-sensitive current. **E** Representative western blots showing the expression of Kir2.1 against CSQ2 (same gel as Fig. 3). **F** Quantification of Kir2.1 expression relative to early hiPSC-CM (3 independent experiments per group). Data are mean ± SEM. *P < 0.05 ***P < 0.001 versus early hiPSC-CM culture. Symbols represent separate differentiations. *n*/*N* = number of hiPSC-CM/differentiation

K^+^ currents are responsible for repolarisation and stabilisation of resting membrane potential (RMP). Assessment of the rapid component of the delayed rectifier K^+^ current (*I*_Kr_) following complete block with 25 µmol/L E-4031 revealed no age-dependent difference in tail current between late and early hiPSC-CM (− 2.94.12 ± 0.55 vs. − 3.84 ± 0.82 pA/pF, *n*/*N* = 77/4 vs. 60/4, *P* = 0.6733; Fig. 5a, b). Comparable I–V curves and pharmacology were also observed across early and late hiPSC-CM (Online Fig. S3).

The basal inward-rectifier K^+^ current *I*_*K*1_ was measured in both early- and late-stage hiPSC-CMs using a modified ramp protocol with high extracellular [K^+^] (20 mmol/L), which produces a positive shift in reversal potential and allows for precise current detection, as previously described [76] (Fig. 5c). Ba^2+^-sensitive *I*_*K*1_ was markedly increased in late-stage cells compared with early cells in both inward direction (− 100 mV: − 48 ± 12.3 vs. − 12.63 ± 2.28 pA/pF, *n*/*N* = 17/4 vs. 43/6, *P* = 0.0004; Fig. 5c, d) and outward direction (− 10 mV: 4.10 ± 0.62 vs. 2.75 ± 0.47 pA/pF, *n*/*N* = 17/4 vs. 43/6, *P* = 0.009), without changes in rectification (Online Fig. S4). In accordance, expression of Kir2.1 protein was significantly increased in late hiPSC-CM cultures (Fig. 5e, f).

### Computational modelling of hiPSC-CM Ca^2+^-handling maturation

**Fig. 6:**
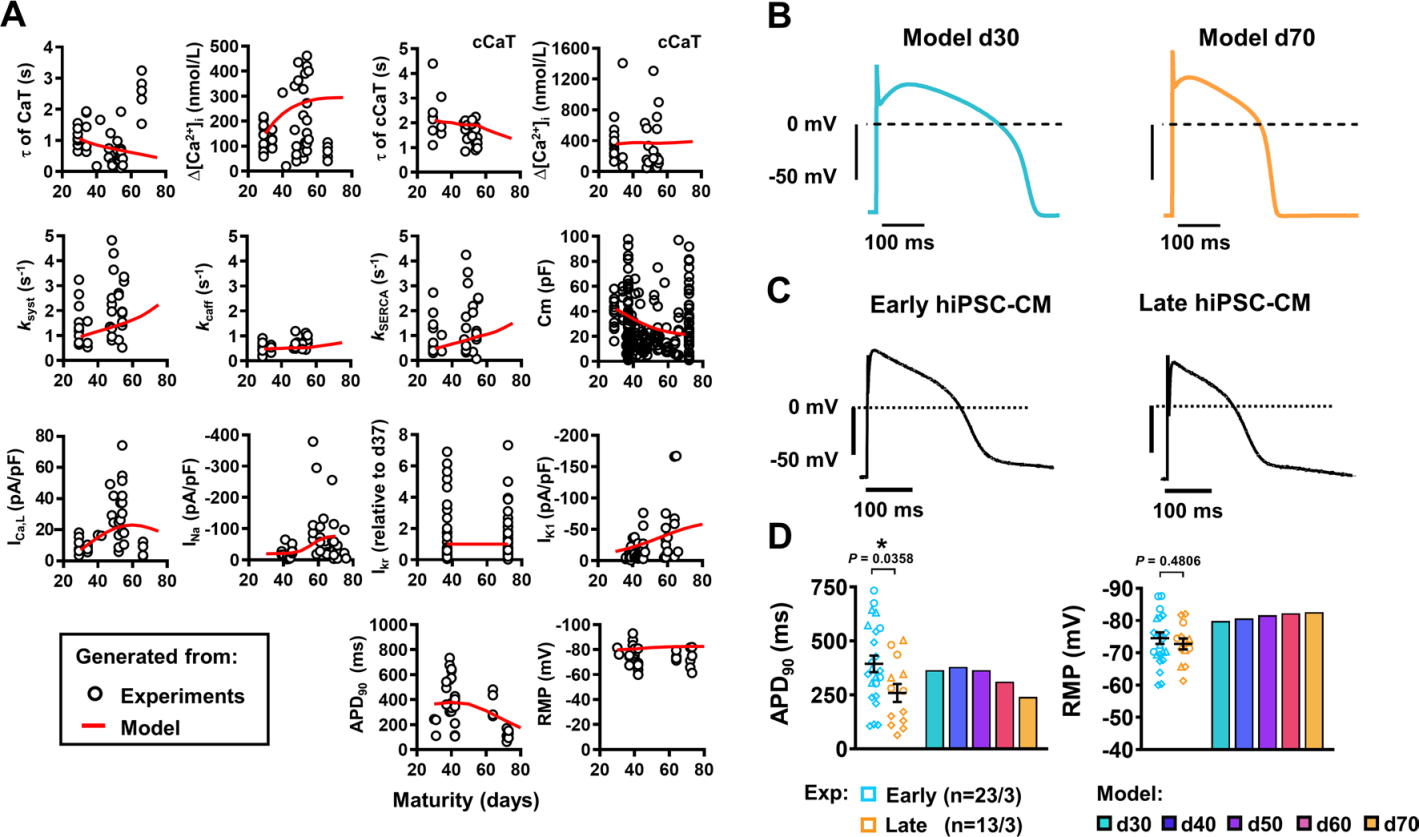
Overview of maturation-dependent changes in cellular Ca^2+^ dynamics and electrophysiology which drive action potential (AP) characteristics in experimental and in silico human induced pluripotent stem cell-derived cardiomyocytes (hiPSC-CM). **A** Plots of the experimental data for all measured electrophysiological cellular parameters in aging hiPSC-CM. The red line indicates the in silico output of expected results demonstrating non-linear maturation-dependent characteristics. **B** Simulated steady-state AP traces at 0.5 Hz in d30 and d70 modelled hiPSC-CM. The stimulus current (I_stim_) was set to − 120 μA/μF and the hyperpolarising current (I_hyper_) was set to 0.2 μA/μF. **C** Representative experimental AP traces in early (left) and late hiPSC-CM (right). **D** Comparison of experimental and model AP properties during 0.5 Hz pacing: resting membrane potential (RMP; left) and AP duration at 90% repolarisation (APD_90_; right). Experimental data are mean ± SEM. *P < 0.05. Symbols represent separate differentiations. *n*/*N* = number of hiPSC-CM/differentiation

We employed computational modelling to assess (1) whether the experimentally identified changes in *I*_Ca,L_, *I*_NCX_ and SERCA are sufficient for the observed changes in hiPSC-CM Ca^2+^-handling and (2) to predict the maturation-dependent changes in AP characteristics resulting from the remodelling of all ionic currents. The recent Kernik et al*.* hiPSC-CM model was fit to our experimental data from early- or late-stage hiPSC-CM [36]. Besides the changes in *I*_Ca,L_, *I*_NCX_ and SERCA function, adjustments in Ca^2+^ buffering and background Ca^2+^ influx were needed to reproduce the experimentally observed Ca^2+^-handling properties (Online Table S2). Nevertheless, with this limited number of changes, the model was able to reproduce all experimentally observed properties of early- and late-stage hiPSC-CM (Online Figs. S4–S8). Of note, interpolation of these Ca^2+^-handling parameters produced non-linear maturation-dependent changes in CaT properties that reflected the non-linear patterns observed in the experimental data (Fig. 6a). These modelling data suggest that gradual increases or decreases in expression levels of Ca^2+^-handling proteins may produce complex temporal changes at the cellular level.

### Identification and experimental corroboration of AP shortening during long-term culture

Under current-clamp conditions at 0.5 Hz, the in silico hiPSC-CM model predicted a maturation-dependent decrease in action potential duration (APD) from 365 ms at d30 to 174 ms at d70. (Fig. 6b). In order to corroborate and validate the maturation-dependent AP shortening predicted by the modelling data, APs were measured at multiple time points during long-term hiPSC-CM monolayer culture (Fig. 6c). Under experimental current-clamp conditions, hiPSC-CM indeed displayed maturation-dependent changes, with late cells showing APD shortening at 50% and 90% repolarisation (APD_50,_ APD_90_) compared with early-stage cells (APD_50_: 163 ± 35.76 vs. 205.34 ± 26.87 ms [not shown]; APD_90_: 259.1 ± 42.13 vs. 393.8 ± 38.65 ms, *n*/*N* = 13/3 vs. 23/3, *P* = 0.0358; Fig. 6d). Repolarisation fraction ([APD_90_-APD_50_]/APD_90_), a representation of repolarisation profile and, therefore, an index of cardiomyocyte subtype, remained unchanged throughout hiPSC-CM culture suggesting the absence of a transient subtype shift during long culture periods (Online Fig. S9). Upstroke velocity and AP amplitude (APA) were increased in late-stage hiPSC-CM (upstroke velocity: 66.98 ± 20.9 vs. 30.22 ± 10.24 mV/ms, *n* = 10/3 vs. 16/3, *P* = 0.0309; APA: 123.7 ± 4.06 vs. 110.6 ± 3.67 mV, *n* = 13/3 vs. 23/3, *P* = 0.0298; Online Fig. S9), consistent with the increase in *I*_Na_. No change was observed between early- or late-stage RMP both in the presence (Fig. 6d) and absence of injected current (Online Fig. S9), which is in line with our in silico simulations. In silico, the unchanged RMP could be attributed to a parallel increase in depolarizing NCX during maturation, counterbalancing the effects of increased *I*_*K*1_ on RMP.

**Fig. 7:**
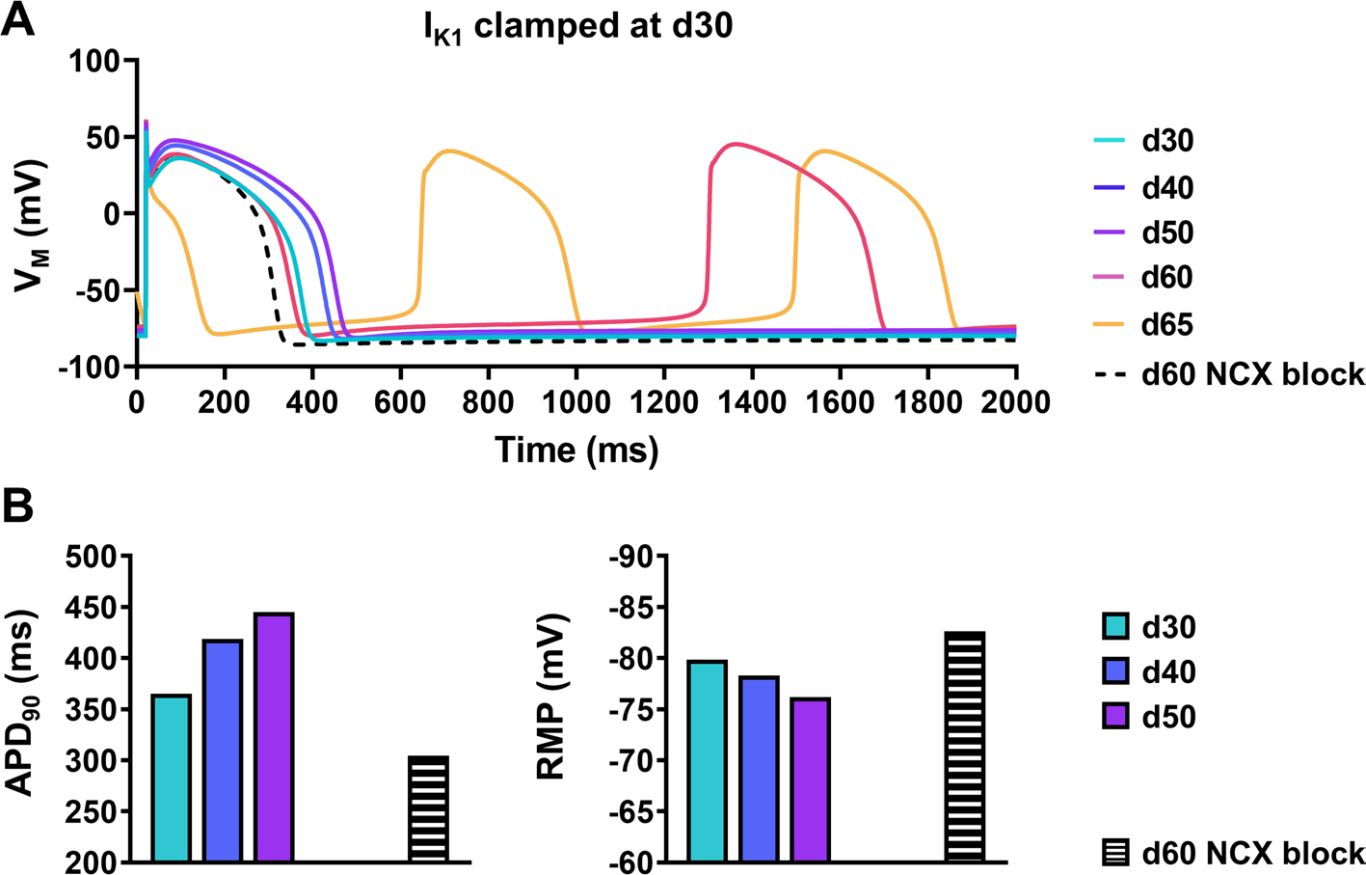
The role of inward-rectifier K^+^ current (*I*_*K*1_) and inward Na^+^/Ca^2+^-exchanger (NCX) current in maturity-dependent action potential (AP) shortening in in silico human induced pluripotent stem cell-derived cardiomyocytes (hiPSC-CM). **A** Steady-state AP simulations over increasing ages with *I*_*K*1_ clamped at 30 days of maturation (solid lines), as well as during acute inhibition of NCX at d60 (dashed line). Note: automaticity is observed at d60 and d65. The stimulated AP at d65 is short due to the incomplete repolarisation of the preceding spontaneous AP. **B** AP duration at 90% repolarisation (APD_90_; left) and resting membrane potential (RMP; right) at increasing stages of development in the absence of *I*_*K*1_ maturation, as well as at d60 with acute NCX inhibition (black/white bars). Note: RMP and APD_90_ values for d60 and d65 in the absence of NCX inhibition are not shown due to abnormal automaticity

We then went back to the model to establish the major ionic determinant of the AP shortening by making use of the perfect control offered by in silico models. Preventing the maturation-dependent increase in *I*_*K*1_ abolished the APD reduction and eventually elicited spontaneous activity (Fig. 7a). A progressive depolarisation of RMP was also observed due to increased NCX (Fig. 7a, b), highlighting the importance of maturation-dependent changes in *I*_*K*1_ for cellular electrophysiology of hiPSC-CM. To further highlight the electrophysiological consequence of increased *I*_*K*1_ in hiPSC-CM experimentally, we evaluated the effects of partial *I*_*K*1_ inhibition. Older cells showed a 50% increase in AP duration following *I*_*K*1_ blockade with BaCl_2_ (1 mmol/L) compared to a non-significant 5% change in early cells (Online Fig. S10). In addition, application of BaCl_2_ produced more instability and a high occurrence of arrhythmogenic events in late cells compared to early hiPSC-CM (Online Fig. S10).

## Discussion

**Fig. 8:**
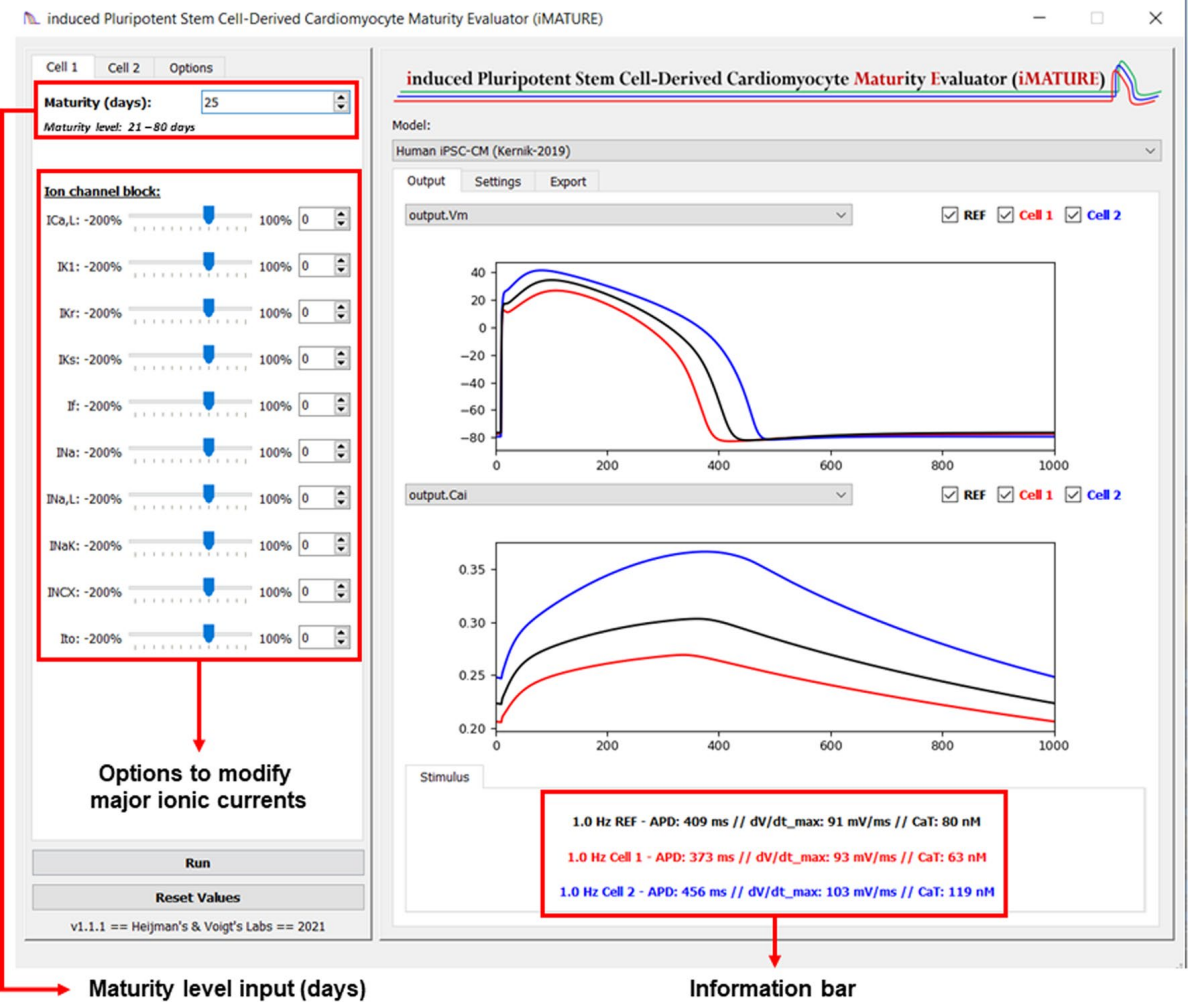
Screenshot of the induced pluripotent stem cell-derived cardiomyocyte maturity evaluator (iMATURE) software tool. The iMATURE tool incorporates the experimentally-observed maturity-dependent changes on cardiac ion channels (i.e., *I*_Na_, I_NaL_, *I*_Ca,L_, *I*_Kr_ and *I*_*K*1_) and Ca^2+^-handling proteins in the Kernik hiPSC-CM model [36]. The tool enables the simulation and comparison of two maturity levels simultaneously under different experimental conditions. It also enables rapid evaluation of the effects of inhibition of major ionic currents

In this multimodal study, we have identified dynamic developmental behaviour of key ionic currents and Ca^2+^-handling properties in hiPSC-CM during long-term culture. Older hiPSC-CM display significantly larger *I*_Ca,L_ density along with temporally complex SERCA and NCX development (Figs. 2, 3). *I*_Na_ and *I*_*K*1_ densities were also significantly increased in late-stage cells, which increases AP upstroke velocity and shortens APD, respectively (Figs. 4, 5, 6; Online Fig. S9). In addition, we updated an existing in silico hiPSC-CM model which reproduced our experimental findings. Interpolation of the model parameters as a function of cellular age revealed complex, nonlinear temporal dynamics of hiPSC-CM electrophysiological development that was consistent with our experimental data (Fig. 6a). Using this tool, we also established the primary ionic determinant of APD shortening by abolishing the maturation-dependent increase in *I*_*K*1_, which successfully attenuated APD reduction (Fig. 7). Finally, we have developed an open-source user interface which allows for multi-level simulations of cellular electrophysiology and Ca^2+^-handling across a wide range of cellular ages post differentiation (Fig. 8). This tool also exhibits the capacity for age-based deductions of drug-induced proarrhythmic risk.

In this study, cardiomyocyte maturity is defined as a general phenotypical state equivalent to that of a fully developed native adult ventricular cardiomyocyte. In particular, this study assesses time-dependent changes in hiPSC-CM calcium handling and electrophysiology as key components of their maturation state. Similar to previous work, our hiPSC-CM show functional variability and an immature electrical phenotype characterized by less negative RMP, slower AP upstroke velocity and automaticity [12, 23, 34]. This is not surprising, as native adult cardiomyocytes develop continually within a complex and precisely organised system over a lifetime of phasic load and physiological stimulation in vivo.

### Maturation of cytosolic Ca^2+^ homeostasis

There is a paucity of systematic studies assessing passive maturation of electrophysiological and Ca^2+^-handling processes in isolated hiPSC-CM cultured for more than 30 days after differentiation onset. Here, we report evidence of robust Ca^2+^-handling and operational SR Ca^2+^ stores in early hiPSC-CM, similar to results reported by Hwang et al*.* [27]. However, as our cells aged further, they displayed increased *I*_Ca,L_ and CaT amplitude (Fig. 2) along with a functional increase in key Ca^2+^-removal mechanisms, NCX and SERCA (Fig. 3). The present work expands on that of Hwang et al*.* and indicates that further maturation of Ca^2+^-handling machinery is possible in hiPSC-CM under prolonged culture of more than 50 days. Previous studies using cells under 30 days of age have outlined a dominant role of NCX function and poor SR development in hiPSC-CM [44]. Increased NCX-mediated electrogenic activity coupled with a leaky SR leads to increased incidence of delayed after-depolarisations (DADs) and could play a role in the cellular automaticity typically displayed by hiPSC-CM. The interplay of low inward-rectifier K^+^ current density paired with increased funny current (*I*_f_) is assumed to be the major cause of spontaneous activity in hiPSC-CMs [66]. However, previous studies have shown that *I*_f_ density alone is not sufficient to induce automaticity in hESC-CM. Instead, Ca^2+^ release from the SR has been hypothesised as a major driver of automaticity with RyR2 abolition leading to cellular quiescence [38]. *I*_*K*1_ also influences cellular automaticity and previous work highlights a potential regulatory effect of increased cytosolic Ca^2+^ flux during diastole by increasing rectification in cardiomyocytes, effectively blocking *I*_*K*1_ and facilitating spontaneous beating [16, 80]. We, among others, report high SR Ca^2+^ leak and increased automaticity in early hiPSC-CM. However, as the cells age, we noted a lower incidence of Ca^2+^ sparks and decreased SR Ca^2+^ leak coupled with a significant decrease in spontaneous beating rate (Online Fig. S2). Interestingly, SR Ca^2+^ load remained comparable between early and late cultures, suggesting coordinated development of Ca^2+^-handling machinery during maturation (Fig. 3). The increased NCX function identified in older hiPSC-CM was not strongly replicated at the protein level, possibly also implicating the concurrent development of intracellular signalling, trafficking and phosphorylation mechanisms. Indeed, increased cAMP and cGMP have been found to enhance forward mode NCX function through protein kinase activation in older, but not in younger, embryonic mouse ventricular cardiomyocytes [58].

Together, these data point to a strong age-dependence of SR function under standardised and prolonged culture conditions. When interpolated in silico, a non-linear behaviour with exponential consolidation of SERCA activity is observed as hiPSC-CM age over time (Fig. 6). Our in silico model is the first to incorporate a detailed analysis of CaT and Ca^2+^-reuptake mechanisms across various stages of hiPSC-CM maturation.

### Maturation of cellular electrophysiology

An overview of hiPSC-CM electrophysiology from 2D and 3D preparations compared with our data and with data from native ventricular cardiomyocytes is provided in Tables 1, 2 and 3. It is important to emphasize that ion currents are dependent on experimental ion concentrations, which differ between studies. Direct comparisons should, therefore, be made with caution, ideally between groups studied under identical conditions. Nevertheless, similar to previous studies, our hiPSC-CM show a relatively immature phenotype characterized by less negative RMP, slower AP upstroke velocity and automaticity [8, 18, 26]. Lack of *I*_*K*1_ is a hallmark of hiPSC-CM [23, 50]. To allow for comparison between studies performed at different extracellular K^+^ concentrations and temperatures, we estimated the conductance values based on reported *I*_*K*1_ densities and calculated the resulting reversal potentials (*E*_rev_, Tables 1, 2,3). The reduced *I*_*K*1_ conductance observed throughout all studies is a major contributor to the less negative RMP and thereby facilitates the occurrence of spontaneous activity in hiPSC-CM constructs [24, 31, 34]. In addition, the depolarized RMP also causes reduced availability of voltage-dependent Na^+^ channels due to incomplete recovery from inactivation. Therefore, the reduced *I*_*K*1_ may also contribute to the typically lower AP upstroke velocity in hiPSC-CM [12, 43, 50]. This combination induces proarrhythmic traits in hiPSC-CM which presents a severe disadvantage for their use for drug safety screening initiatives such as CiPA. Targeting *I*_*K*1_ is therefore an important aspect of enhancing hiPSC-CM maturity and several methods exist to increase inward rectifier density. The hybrid method of dynamic patch clamp aims to overcome this limitation using an in silico ion channel model to adjust hyperpolarising current injection in real time based on the measured membrane potential [18, 52]. This technology is promising; however, it cannot capture native regulation of *I*_*K*1_, e.g., by intracellular Ca^2+^ or Na^+^ [22, 75, 80]. Our combination of standardised serum-free differentiation and prolonged monolayer culture of > 50 days is sufficient to produce a 70% increase in *I*_*K*1_ density with increased *I*_Na_ and concomitant increases in AP upstroke velocity (Figs. 4, 5, Online Fig. S9). Similar maturation-dependent increases in *I*_*K*1_ density have been reported previously in hESC-CMs during prolonged culture [37, 64]. Doss et al*.* reported a transient increase in *I*_*K*1_ density in hiPSC-CMs after 2 months, followed by a decrease after 4 months [12]. Our in silico extrapolation of *I*_*K*1_ maturation does not support this parabolic developmental pattern, which could be due to differences in cellular culture techniques or their harsher dissociation procedures prior to measurement.

**Table 1:** Comparison of electrophysiological properties of human induced pluripotent stem-cell derived cardiomyocytes

	This study, “Early”	This study, “Late”	Zhao et al. [81]	Zhao et al. [81]	Ma et al. [50]	Lee et al. [43]
Capacitance (pF)	43	31	22–25	22–25	16	
*I*_Na_						
Peak I_Na_ density	− 27	− 71	− 30	− 53	− 217	− 163
Potential	− 25	− 25	− 40	− 40	− 20	− 60
[Na]_e_ for Peak *I*_Na_ (mM)	5	5	10	10	10	5
[Na]_e_ for Peak* I*_Na_ (mM)	5	5	20	20	50	50
Temperature (°C)	37	37	20	20	36	37
*E*_rev_ (mV)	0	0	18	18	43	62
Conductance (S/F)	1065	2845	522	918	3447	1343
*I*_Ca,L_						
*I*_Ca,L_ density	− 11	− 29	− 10	− 13	− 17	− 7
Temperature (°C)	37	37	22–25	22–25	22–25	37
*I*_K1_						
*I*_K1_ density (pA/pF)	− 13	− 48	− 1	− 2	− 2	− 5
Step potential	− 100	− 100	− 120	− 120	− 123	− 150
[K]_i_ for* I*_K1_ (mM)	150	150	126	126	150	140
[K]_e_ for* I*_K1_ (mM)	20	20	6	6	5	5
Temperature (°C)	37	37	20	20	37	37
*E*_rev_ (mV)	− 54	− 54	− 77	− 77	− 89	− 87
Conductance (S/F)	274	1040	16	45	67	81
*I*_Kr_						
*I*_Kr_ tail (pA/pF)	− 4	− 3				
*I*_Kr_ step (pA/pF)			1	1	0.4	2
Temperature (°C)	20	20	20	20	37	37
Action Potential						
RMP (mV)	− 75	− 73	− 80	− 80	− 76	− 66
dV/dt_max_ (V/s)	30	67	125	130	28	45
APA (mV)	110	123	140	140	104	108
APD_90_ (ms)	394	259	290	300	415	492
Frequency	0.5	0.5	1	1	Spontaneous	Spontaneous
Temperature (°C)	37	37	20	20	37	37
Days after differentiation onset	30–46	47–80	30–40	50–60	10–21*	7–28*

	Veerman et al. [71]**	Herron et al. [23]	Sala et al. [63]	Doss et al. [12]	Lemoine et al. [46]	Horváth et al. [25]
Capacitance (pF)					28	31
*I*_Na_						
Peak* I*_Na_ density	− 94	− 105			− 10	
Potential	− 20	− 30			− 30	
[Na]_i_ for Peak* I*_Na_ (mM)	3	5			5	
[Na]_e_ for Peak* I*_Na_ (mM)	20	20			5	
Temperature (°C)	20	20			20	
*E*_rev_ (mV)	48	35			0	
Conductance (S/F)	1378	1615			343	
*I*_Ca,L_						
*I*_Ca,L_ density	− 50		− 9			
Temperature (°C)	36		37			
*I*_K1_						
*I*_K1_ density (pA/pF)		− 6		− 3		− 33
Step potential		− 120		− 100		− 100
[K]_i_ for* I*_K1_ (mM)		148		120		150
[K]_e_ for* I*_K1_ (mM)		5		5		20
Temperature (°C)		20		36		37
*E*_rev_ (mV)		− 84		− 83		− 54
Conductance (S/F)		173		201		717
*I*_Kr_						
*I*_Kr_ tail (pA/pF)			2	1		
*I*_Kr_ step (pA/pF)						
Temperature (°C)			37	37		
Action Potential						
RMP (mV)	− 80**	− 71	− 48	− 66		
dV/dt_max_ (V/s)	230	147		24		
APA (mV)	120	116	90	103		
APD_90_ (ms)	160	NA	135	325		
Frequency	1		1	Spontaneous		
Temperature (°C)	37	37	37	37		
Days after differentiation onset	39–44	4–7*	20–30	35–74	42	42

Currents are dependent on ion concentrations, which differ between studies. Be cautious with direct comparisons which should ideally be made between groups studies under identical conditions

*Days post thaw

**Dynamic clamp

**Table 2:** Electrophysiological properties of hiPSC-CM in engineered heart muscle

	Lemoine et al. [46]	Lemoine et al. [45]	Horváth et al. [25]	Tiburcy et al. [68]
Capacitance (pF)	28		47	
*I*_Na_				
Peak* I*_Na_ density	− 19			
Potential	− 30			
[Na]_i_ for Peak* I*_Na_ (mM)	5			
[Na]_e_ for Peak* I*_Na_ (mM)	5			
Temperature (°C)	20			
*E*_rev_ (mV)	0			
Conductance (S/F)	633			
*I*_Ca,L_				
*I*_Ca,L_ density				
Temperature (°C)				
*I*_K1_				
*I*_K1_ density (pA/pF)			− 14	
Step potential			− 100	
[K]_i_ for* I*_K1_ (mM)			150	
[K]_e_ for* I*_K1_ (mM)			20	
Temperature (°C)			20	
*E*_rev_ (mV)			− 51	
Conductance (S/F)			285	
*I*_Kr_				
*I*_Kr_ tail (pA/pF)				
*I*_Kr_ step (pA/pF)				
Temperature (°C)				
Action Potential				
RMP (mV)	− 78	− 78	− 75	− 72
dV/dt_max_ (V/s)	219	348		107
APA (mV)	103	109		97
APD_90_ (ms)	NA	255	271	436
Frequency (Hz)	1	1	1	Spontaneous
Temperature (°C)	37	37	37	37
Days after differentiation onset	42	39–114	42	NA

Currents are dependent on ion concentrations, which differ between studies. Be cautious with direct comparisons which should ideally be made between groups studies under identical conditions

**Table 3:** Electrophysiological properties of native human ventricular cardiomyocytes

	Sakakibara et al. [61]	Valdivia et al. [69]	Magyar et al. [51]**	Mewes et al. [53]	Konarzewska et al. [41]	Koumi et al. [42]*
Capacitance (pF)	194	168		189	96	
*I*_Na_						
Peak* I*_Na_ density	− 20	− 49				
Potential	− 40	− 40				
[Na]_i_ for Peak* I*_Na_ (mM)	5	1				
[Na]_e_ for Peak* I*_Na_ (mM)	5	5				
Temperature (°C)	17	20				
*E*_rev_ (mV)	0	41				
Conductance (S/F)	505	608				
*I*_Ca,L_						
*I*_Ca,L_ density			10	4		
Temperature (°C)			37	21–23		
*I*_K1_						
*I*_K1_ density (pA/pF)			− 4		− 18	− 30
Step potential			− 100		− 140	− 120
[K]_i_ for* I*_K1_ (mM)			120		525	141
[K]_e_ for* I*_K1_ (mM)			5		4	5
Temperature (°C)			37		20	37
*E*_rev_ (mV)			− 83		− 123	− 87
Conductance (S/F)			210		1083	914
*I*_Kr_						
*I*_Kr_ tail (pA/pF)			0			
*I*_Kr_ step (pA/pF)						
Temperature (°C)			37			
Action Potential						
RMP (mV)			− 82			
dV/dt_max_ (V/s)			215			
APA (mV)			107			
APD_90_ (ms)			213			395
Frequency			1			1
Temperature (°C)			37			37
Heart failure patients (+/−)	+	−	−	−	−	−

	Bailly et al. [2]	Iost et al. [28]	Hartmann et al. [20]*	Lemoine et al. [46]**	Lemoine et al. [45]**
Capacitance (pF)					
*I*_Na_					
Peak* I*_Na_ density					
Potential					
[Na]_i_ for Peak* I*_Na_ (mM)					
[Na]_e_ for Peak* I*_Na_ (mM)					
Temperature (°C)					
*E*_rev_ (mV)					
Conductance (S/F)					
*I*_Ca,L_					
*I*_Ca,L_ density					
Temperature (°C)					
*I*_K1_					
*I*_K1_ density (pA/pF)	− 60				
Step potential	− 170				
[K]_i_ for* I*_K1_ (mM)	120				
[K]_e_ for* I*_K1_ (mM)	4				
Temperature (°C)	20				
*E*_rev_ (mV)	− 86				
Conductance (S/F)	714				
*I*_Kr_					
*I*_Kr_ tail (pA/pF)		0.3			
*I*_Kr_ step (pA/pF)					
Temperature (°C)		37			
Action Potential					
RMP (mV)			− 80	− 75	− 78
dV/dt_max_ (V/s)			182	253	176
APA (mV)			130	105	111
APD_90_ (ms)			553	NA	397
Frequency			1	1	1
Temperature (°C)			37	37	37
Heart failure patients (+/−)	−	−	+	+	+

Currents are dependent on ion concentrations, which differ between studies. Be cautious with direct comparisons which should ideally be made between groups studies under identical conditions

*Action potentials from isolated cardiomyocytes

**Action potentials from intact tissue preparations

In the majority of studies, the maximal peak *I*_Na_ amplitude appears to be larger in hiPSC-CM compared with native cardiac tissue (Tables 1, 2, 3). This contradicts the lower expression levels of the underlying Nav1.5 subunit which are found in hiPSC-CM in comparison to human native ventricular cardiomyocytes [7]. Furthermore, direct comparison of peak *I*_Na_ currents is often hindered by different experimental conditions such as extracellular Na^+^ concentration and temperature. A direct comparison of hiPSC-CM and native human ventricular cardiomyocytes by Lemoine et al*.* suggests that peak *I*_Na_ currents are indeed lower in hiPSC-CM [46]. In addition, Lemoine et al. provide evidence for higher peak *I*_Na_ currents in more advanced 3D tissue culture models. This is in agreement with our study and a previous study in hiPSC-CM showing a tendency towards increased peak Na^+^ current in older hiPSC-CM [81]. Taken together, it appears that electrophysiological maturation of hiPSC-CM is associated with increased *I*_Na_ densities, although AP upstroke velocity remains low compared to adult ventricular cardiomyocytes.

The present work identifies maturation-dependent *I*_*K*1_ augmentation as a key mediator of AP shortening in hiPSC-CM. Rapid delayed rectifier K^+^ currents remained unchanged throughout long term culture (Fig. 5) and, in the absence of maturation-dependent changes of *I*_*K*1_ in silico, APD prolonged as the cells matured in the presence of increasing cytosolic Ca^2+^ activity (Fig. 7). This is in contrast to the Paci model [54], which attributes maturation-dependent AP shortening of hiPSC-CM to *I*_Ca,L_ and *I*_Kr_ dynamics. AP shortening upon *I*_*K*1_ injection has indeed been shown previously in native cardiomyocytes [72], in hiPSC-CMs [4, 70], and in silico models [14]. To our knowledge, we provide the first evidence of maturation-induced *I*_*K*1_ mediation of APD shortening in hiPSC-CM. This finding, along with our accompanying iMATURE maturation simulation software, could provide insight into the heterogeneous AP profiles which are regularly reported within and between hiPSC-CM cohorts. Future innovative studies promoting hiPSC-CM maturation should focus similarly on *I*_*K*1_ development as this will be a key component in optimising hiPSC-CM for widespread screening initiatives [25].

### Potential implications

Inherent variability in hiPSC-CM function hinders reliable quantification of average behaviour. In silico modelling has emerged as a powerful solution to link dispersed data sets and precisely define cellular parameters that contribute to experimentally observed heterogeneity. The recent state-of-the-art Kernik hiPSC-CM model integrates a wide range of experimental data by building a predictive array of cellular variability that allows for detailed investigation of cellular electrophysiology and underlying causal mechanisms of phenotypical heterogeneity [36]. Despite the availability of several useful hiPSC-CM models, none has comprehensively considered the ages of the hiPSC-CM used in the underlying experimental data. Using our own experimental findings and the framework of the Kernik model, we present the first hiPSC-CM model in which minimal parameter change can reproduce a wide range of electrophysiological properties of early- and late-stage hiPSC-CM (Online Figs. S4–S8). To facilitate analyses of maturation-dependent effects, our model has been integrated into an open-source interface, iMATURE, which allows the user to manually select the age of hiPSC-CM post differentiation and receive predictive readouts of AP morphology and ion channel dynamics over their specified age range. This platform also allows for manual modulation of individual ion channels and Ca^2+^ fluxes at any cellular age between 21 and 80 days post differentiation, enabling the investigation of age-dependent responses to hypothetical cardiotropic compounds (Fig. 8). Increased understanding of the impact of time-dependent phenotypic changes in hiPSC-CM is expected to contribute to standardisation of methodological techniques. Accordingly, this may enhance the quality of hiPSC-CM platforms, reduce variability in functional readouts, and promote efficient outlets for personalised medicine and streamline drug development [21].

### Potential limitations

In addition to prolonged cultivation times, strategies to enhance hiPSC-CM maturity include the adoption of appropriate cardiac differentiation protocols [6], culture substrates [23] and the application of mechanical, chemical or electrical stress to hiPSC-CM embedded in 3D hydrogels or fibrin blocks to more closely replicate the in vivo environment [35, 57, 68]. From an electrophysiological point of view, 2D cultivation strategies seem to be particularly limited with respect to the slow upstroke velocity of the AP, which is a common finding in all studies (Table 1). In contrast, 3D culture strategies seem to provide a promising improvement showing faster AP upstroke velocities (Table 2) [46]. It is important to note that the concept of hiPSC-CM maturation is a broad paradigm which also encompasses molecular, metabolic and structural properties [47]. Previous reports of hiPSC-CM maturation indeed show structural elongation and heightened sarcomere organisation, resembling the classical rod shape of cardiomyocytes [33, 49]. We observed highly heterogeneous morphological features in our developing cellular cultures with both rod-like and rounded cells. Measuring individual cells in sparsely seeded monolayers is essential for gathering true electrophysiological readouts; however, this environment is inherently artificial due to severely decreased cell-to-cell communication. Therefore, various external paracrine effects and electrical stimuli may not play a major role in the development of our cells [37]. Our differentiation technique enables a baseline assessment of cellular function under fully defined culture conditions. Further studies using targeted maturation-enhancing techniques can, therefore, build upon this foundation.

In addition to the remodelling characterised in the present study, other currents may contribute to hiPSC-CM electrical development and age-dependent action potential shortening. Cardiac currents such as *t*-type calcium current (*I*_Ca,T_), *I*_f_, transient-outward K^+^ current (*I*_to_) or the slow component of the delayed-rectifier K^+^ current (*I*_Ks_) were not experimentally quantified, although they are present within the Kernik hiPSC-CM computer model [36] that our work is based on. Previous studies have identified a large role for *I*_Ks_ and *I*_Kr_ in the maintenance of repolarization reserve in adult human cardiomyocytes [5, 32]. During our experimental examination of *I*_*K*1_ block on AP duration, we cannot exclude the possibility that our high concentration of BaCl_2_ could also cause unspecific inhibition of the delayed-rectifier K^+^ currents [32]. Following consolidation with our entire experimental data set, our iMATURE platform is able to provide appropriate readouts of all cardiac-related currents and does not indicate a strong contribution of the delayed-rectifier K^+^ currents in age-related AP shortening in hiPSC-CM.

hiPSC-CMs also show regional subtype-specific traits that allow them to be classified as ventricular-, atrial- or nodal-like cardiomyocytes [29]. In the absence of an intervention to direct subtype differentiation, for example by promoting an atrial phenotype with retinoic acid, hiPSC-CM generally show predominantly ventricular traits, with minimal mixing of other subtypes [10]. We cannot definitively state that our experimental data contains only ventricular-like cells. Differences in ionic makeup of atrial and nodal cells influence their AP morphology and repolarisation profile, which are, therefore, commonly utilised as functional markers of cellular subtype [13]. The repolarisation fraction provides an index of phase 3 AP kinetics and, in our hands, does not show evidence of atrial or nodal cell contamination in our experimental cohort. In addition, no significant change in repolarisation fraction was detected during maturation, indicating the absence of a subtype-shift during prolonged culture as has been recently proposed [3].

Additionally, some potential limitations should be considered related to the computational modelling performed in this study. (1) Although we employed a widely used and well-validated state-of-the-art in silico model of hiPSC-CM [36], the model dependence of our results cannot be excluded. (2) The intercellular heterogeneity of ion channel distribution and activity could potentially affect our observed AP properties. Here, we present a single deterministic hiPSC-CM model without intercellular variability. Although we do identify an overall trend of age-dependent AP shortening in hiPSC-CM, future research should employ high-throughput electrophysiological techniques and multiple populations of in silico hiPSC-CM models to properly capture and map this phenomenon.

## Conclusions

In this study we have shown that hiPSC-CM under standard and simplified culture conditions show distinct alterations in electrical function and Ca^2+^ handling over extended time periods. Key ionic currents such as *I*_Ca,L_ and *I*_*K*1_, crucial for homeostatic cellular function, show increased functional expression in older hiPSC-CM cultures, with the latter likely contributing to maturation-dependent AP shortening. Our experimental data fit well within established in silico frameworks, and our new user interface software allows for easy and rapid analysis of an optimal temporal ‘window’ in which disease modelling or assessments of proarrhythmic risk can be effectively performed, thus minimising heterogeneity between functional readouts.

### Supplementary Information

Below is the link to the electronic supplementary material.

Supplementary file 1 (PDF 1801 KB)

## Data Availability

All available data are incorporated into this article and its online supplementary material. iMATURE and Myokit are both freely available and can be downloaded from the authors’ websites (www.github.com/jordiheijman and http://www.myokit.org). Detailed installation instructions are provided in the Online Supplement.
