# Supplementary material for: Electrophysiological and calcium-handling development during long-term culture of human-induced pluripotent stem cell-derived cardiomyocytes
Source: Basic Res Cardiol. 2023 Apr 5;118(1):14. doi: 10.1007/s00395-022-00973-0 (PMC10076390; doi:10.1007/s00395-022-00973-0)
Supplement: Supplementary file 1 — (PDF 1801 KB) [file 395_2022_973_MOESM1_ESM.pdf]

Electrophysiological and calcium-handling development during long-term culture of human induced pluripotent stem cell-derived cardiomyocytes

Fitzwilliam Seibertz, MSc*, Henry Sutanto, MD, PhD*, Rebekka Dülk, MD, Julius Ryan D. Pronto, MSc, Robin Springer, Markus Rapedius, PhD, Aiste Liutkute, MSc, Melanie Ritter, Philipp Jung, Lea Stelzer, Luisa M. Hüsgen, Marie Klopp, Tony Rubio, MSc, Funsho E. Fakuade, PhD, Fleur E. Mason, PhD[1,2], Nico Hartmann, MD, Steffen Pabel, MD, Katrin Streckfuß-Bömeke, PhD, Lukas Cyganek, PhD, Samuel Sossalla, MD, Jordi Heijman, PhD✉, Niels Voigt, MD✉

Online data supplement

*These authors contributed equally to this work

Correspondence to:
Dr. Niels Voigt,
Institute of Pharmacology and Toxicology,
Robert-Koch-Straße 40, 37075 Göttingen,
Germany
Phone: 00495513965174;
Fax: 00495513965169;
E-mail: niels.voigt@med.uni-goettingen.de

Dr. Jordi Heijman,
Department of Cardiology, Maastricht University,
Universiteitssingel 50,
6229 ER Maastricht,
The Netherlands
Email: jordi.heijman@maastrichtuniversity.nl

Table of Contents

Experimental Methods **3**
hIPSC reprogramming and culture 3
Directed cardiac differentiation 4
Measurement of basal inward-rectifier K^+ current (I_{K1}) 5
Measurement of early and late Na^+ current (I_{Na}, $I_{Na,L}$) 5
Measurement of the rapid component of the delayed rectifier current (I_{Kr}) 6
Simultaneous measurement of intracellular [Ca^{2+}] and $I_{Ca,L}$ 7
Quantification of SERCA activity. 9
Action potential (AP) recordings 10
Western Blot 11
Flow Cytometry 11
Immunocytochemical staining 12
Computational modeling 13

Supplemental Tables **16**

Supplemental Figures **18**

References **28**

Experimental Methods

hIPSC reprogramming and culture

The human induced pluripotent stem cell (hiPSC) line UMGi014-C clone 14 (isWT1.14) was derived from dermal fibroblasts of a healthy male donor. They were cultured in feeder-free conditions using the integration-free CytoTune iPS 2.0 Sendai Reprogramming Kit (Thermo Fisher Scientific) with reprogramming factors OCT4, KLF4, SOX2, c-MYC.

All protocols for biopsy procurement, and somatic cell reprogramming and differentiation were approved by the ethics committee of the University Medical Center Göttingen (10/9/15).

hiPSC were maintained in feeder-free and growth-factor free conditions with Stem MACS IPS-Brew XF medium (Miltenyi Biotec) refreshed daily at 37°C. Cells were cultured in 1:120 Matrigel™ (BD Biosciences) coated 6-well plates and passaged every 3-4 days upon reaching confluence. During passaging, cells were briefly washed with versene (Thermo Fischer Scientific), then incubated with fresh versene for 2-3 minutes. 1 ml of fresh Stem MACS medium was then used to flush each well to ensure full monolayer detachment. Cells were counted using a CASY counter. For continuing expansion cultures, fresh Matrigel™ coated 6-well plates were filled with new Stem MACs solution supplemented with 1:2000 ROCK inhibitor Y27632 (Stemolecule). Roughly 1-2 million cells were added to each well prior to incubation and continued daily maintenance with Stem MACS at 37°C.

Directed cardiac differentiation

After final passaging, hiPSCs cultured in two-dimensional monolayers were left for 3 days to reach 70-100% confluence. Differentiation begins on day 0 (d0) with the 48 hour application of 4-6 µM CHIR99021 (Sigma-Aldrich) in a 'Differentiation Medium' containing: RPMI 1640 with Glutamaxx (Thermo Fisher Scientific), 0.5 mg/ml human recombinant albumin and 0.2 mg/ml L-ascorbic acid 2-phosphate (all Sigma-Aldrich). Subsequent application of 2.5 µM IWP2 (Sigma-Aldrich) for a further 48 hours stimulates WNT signalling cessation. Contraction was observed around d7. At d8 medium was changed to a 'Culture Medium' containing: RPMI 1640 with Glutamax, and 2% B27 (Thermo Fisher Scientific). Cardiomyocyte purification by lactate selection was performed between d15 and d20 with a 'Selection Medium' containing RPMI 1640 without glucose (Thermo Fisher Scientific), 0.5 mg/ml human recombinant albumin, 0.2 mg/ml L-ascorbic acid 2-phosphate, and 4 mM lactate (all Sigma-Aldrich). Subsequently, hiPSC-CM were maintained with culture medium every 2-3 days. Between d27 and d30, purified iPSC-CMs were digested with TrypLE (Thermo Fisher Scientific) and seeded on round borosilicate glass 10 mm diameter #0 coverslips at a density of 15,000 cells/cm^{-2}. Coverslips were incubated in culture medium with 1:2000 ROCK inhibitor Y27632 (Stemolecule) 24 hours. Subsequently, medium was changed to pure culture medium and refreshed every 2-3 days.

Measurement of basal inward-rectifier K^+ current (I_{K1})

Measurement of I_{K1} was performed using the whole-cell ruptured-patch configuration. Borosilicate glass pipettes gave tip resistances of 2-5 MΩ when filled with pipette solution containing (in mmol/L): EGTA 0.02, GTP-Tris 0.1, HEPES 10, K-aspartate 92, KCl 48, Mg-ATP 1, Na_2-ATP 4; pH = 7.2. Seal resistances were 3-6 GΩ.

The coverslip was superfused with a bath solution at 37°C containing (mmol/L): NaCl 120, KCl 20, $MgCl_2$ 1, $CaCl_2$ 2, glucose 10, HEPES 10; pH = 7.4. A high (20 mmol/L) extracellular K^+ concentration depolarises the I_{K1} reversal potential and allows for in-depth dissection of inward currents at -100mV. Basal current was measured in voltage-clamp configuration at 0.5 Hz using a protocol with a holding potential at -80mV followed by a depolarising ramp pulse from −100 to +40 mV. I_{K1} was identified as the current responsive to Ba^{2+} application (1-mmol/L). Signals were amplified with an Axopatch 200B microelectrode amplifier and acquired and analysed using pClamp-Software V 10.7 (both from Axon Instruments Inc., Foster City, USA). Membrane currents were corrected for membrane capacitance (a measure of cell size) and expressed in pA/pF.

Measurement of early and late Na^+ current (I_{Na}, $I_{Na,L}$)

Measurement of I_{Na} was performed using the whole-cell ruptured-patch configuration. Borosilicate glass pipettes gave tip resistances of 2-5 MΩ when filled with pipette solution containing (in mmol/L): NaCl 5, EGTA 10, GTP-Tris 0.4, HEPES 10, Mg-ATP 4, CsCl 20, $CaCl_2$ 3, Cs-Methansulfonate 90; pH = 7.2. Seal resistances were 3-6 GΩ.

The coverslip was superfused with a bath solution at room temperature containing (mmol/L): NaCl 5, HEPES 10, $MgCl_2$ 1, CsCl 10, glucose 10, $CaCl_2$ 0.5, and TEA-Cl 120 (pH = 7.4, adjusted with CsOH). I_{Na} was measured in voltage-clamp configuration at 0.5 Hz using a protocol with a holding potential at -80mV, followed by a 100 ms step to -110 mV to increase the availability of Na^+ channels. 30 ms steps from -80 to +20 mV were used to elicit channel opening to generate current-voltage (I-V) curves. Peak I_{Na} was identified as current that was blocked by tetracaine, as described by Poulet *et al*. [4]. $I_{Na,L}$ was measured by restoring 120 mmol/L NaCl to the bath solution with a voltage-clamp protocol holding at -120 mV, followed by a 5 ms activating step to +50 mV (in order to maintain voltage control) before a 300 ms pulse at -30 mV as suggested by Sosalla *et al.* [6]. Both I_{Na} and $I_{Na,L}$ were recorded in the presence of Nifedipine (1 µmol/L). $I_{Na,L}$ was identified as tetrodotoxin-sensitive current and the integral of the current was measured between 50 and 250 ms after the -30 mV pulse. Signals were amplified with an Axopatch 200B microelectrode amplifier and acquired and analysed using pClamp-Software V 10.7 (both from Axon Instruments Inc., Foster City, USA). Membrane currents were corrected for membrane capacitance (a measure of cell size) and expressed in pA/pF.

Measurement of the rapid component of the delayed rectifier current (I_{Kr})

Measurement of I_{Kr} was performed using ruptured whole cell configuration in a high performance automated patch clamp platform (SyncroPatch 384; Nanion Technologies GmbH). Cells were isolated using the same TrypLE process defined above and stored in suspension in the SyncroPatch cell hotel prior to measurements.

hiPSC-CM were measured at room temperature using partial borosilicate glass, single-aperture, 384-well planar chips (NPC384T 1x S-type). Seal resistance, series resistance and cell capacitance were acquired from each well via a test pulse. PatchControl 384 (Nanion Technologies GmbH) software digitized and acquired the data. A voltage clamp protocol consisting of a holding potential of -80 mv followed by a 2 s step to 60 mV with steps of 10 mV was employed for I-V acquisition as described previously [3]. External solution contained (in mmol/L): CsCl 144, $CaCl_2$ 2, $MgCl_2$ 2, glucose 5, HEPES 10 (pH = 7.4 adjusted with CsOH). Internal solution contained (in mmol/L) CsCl 20, EGTA 10, HEPES 10, CsF 110 (pH = 7.2 adjusted with CsOH) in accordance with previous protocols [3]. Cs^+ was used as a charge carrier due to its selectivity for the hERG channel. After measurement of I_{Kr} in reference external solution, 25 µmol/L E-4031 was added to block hERG channels and ascertain the molecular basis of the current. Tail current amplitude was analysed using DataControl 384 software (Nanion Technologies GmbH). Membrane currents were corrected for membrane capacitance (a measure of cell size) and expressed as pA/pF.

Simultaneous measurement of intracellular [Ca^{2+}] and $I_{Ca,L}$

Quantification of intracellular [Ca^{2+}] was achieved by loading a single coverslip of iPSC-CM with 10 µmol/L Fluo-3-acetoxymethyl ester (Fluo-3-AM, Invitrogen; 10 min loading and 30 min de-esterification) under low light conditions. Simultaneously, L-type Ca^{2+} currents ($I_{Ca,L}$) were recorded in whole-cell ruptured-patch configuration. Borosilicate glass pipettes gave tip resistances of 2-5 MΩ when filled with pipette solution containing (in mmol/L): EGTA 0.02, Fluo-3 pentapotassium salt 0.1 (Invitrogen), GTP-Tris 0.1, HEPES 10, K-aspartate 92, KCl 48, Mg-ATP 1, Na_2-ATP 4; pH=7.2. Seal resistances were 3-6 GΩ.

On the heated (37°C) stage of an inverted epifluorescence microscope, the iPSC-CM coverslip was superfused with a bath solution containing (in mmol/L): 4-aminopyridine 5, $BaCl_2$ 0.1, $CaCl_2$ 2, glucose 10, HEPES 10, KCl 4, $MgCl_2$ 1, NaCl 140, probenecid 2; pH = 7.35. 4-aminopyridine and $BaCl_2$ were added to block K^+ currents. Nifedipine (1 µmol/L, 10 µmol/L, All Sigma Aldrich) was applied via a rapid solution exchange-system (ALA Scientific Instruments, Long Island, USA). Caffeine (10 mmol/L) was applied for rapid depletion and measurement of sarcoplasmic reticulum (SR) Ca^{2+} content. $I_{Ca,L}$ was measured at 0.5 Hz in voltage-clamp configuration using a protocol with a holding potential at -80 mV and a 100 ms ramp pulse to -40 mV (inactivating fast Na^+ current) followed by a 100 ms test-pulse to +10 mV triggering the subsequent $[Ca^{2+}]_i$ transients. I-V curves were measured by altering the test pulse from -40 mV by 5 mV every 2 seconds with a final pulse at 60 mV. All electrophysiological signals were amplified with an Axopatch 200B microelectrode amplifier and acquired and analysed using pClamp-Software V 10.7 (both from Axon Instruments Inc., Foster City, USA). Membrane currents were corrected for membrane capacitance (a measure of cell size) and expressed in pA/pF.

Fluo-3 fluorescence was excited with a 470 nm LED was collected with a 535/50 emission filter with a photomultiplier mounted into an Optoscan system (Cairn Research, Kent, UK) optimised for high-speed signal capture (10 kHz). Emission was correlated to $[Ca^{2+}]_i$ with the following formula.

$$[Ca^{2+}]_i = k_d \cdot \frac{F}{(F_{max} - F)}$$

Here, K_d represents the dissociation constant of Fluo-3 (864 nmol/L), F denotes Fluo-3 fluorescence, and F_{max} describes Ca^{2+} saturated fluorescence which is collected through cellular laceration at the end of each experiment. Ca^{2+} sparks in Fluo-3 loaded hiPSC-CMs were captured in separate experiments using an LSM 5 Pascal Laser module confocal microscopy system (Carl Zeiss, Jena, Germany) with a 40× oil objective in line-scan mode (512 pixels, 37.5 μm, 1302 Hz, 10,000 cycles, pinhole 67 μm) and Zen 2009 acquisition software.

Quantification of SERCA activity.

Ca^{2+} reuptake into the SR mediated by SERCA, and Ca^{2+} extrusion into extracellular space by the Na^+/Ca^{2+} exchanger (NCX, forward-direction) contribute to the decay (τ) of the $I_{Ca,L}$-induced Ca^{2+} -transient (CaT). The rate constant of decay (k_{syst}) describes the rate of combined Ca^{2+} transport by SERCA and NCX and can be expressed as the sum of the two separate rate constants:

$$k_{syst} = k_{SERCA} + k_{NCX}$$

The decay (τ) of caffeine-induced CaT (cCaT) is almost exclusively due to Ca^{2+} removal through NCX, and can be estimated by analysing the rate of decay of the cCaT (K_{caff}):

$$k_{caff} = k_{NCX}$$

SERCA activity can therefore be estimated by:

$$k_{SERCA} = k_{syst} - k_{NCX}$$

Action potential (AP) recordings

For an experiment, a single coverslip was inserted onto the warmed stage (37°C) of an inverted microscope. APs were recorded in current-clamp mode using the whole-cell ruptured-patch configuration. High resistance borosilicate glass pipettes gave tip resistances of 5-10 MΩ when filled with pipette solution containing (in mmol/L): EGTA 0.02, GTP-Tris 0.1, HEPES 10, K-aspartate 92, KCl 48, Mg-ATP 1, Na_2-ATP 4; pH = 7.2. The coverslip was superfused with a bath solution at 37°C of modified Tyrode's solution containing (in mmol/L): $CaCl_2$ 2, glucose 10, HEPES 10, KCl 4, $MgCl_2$ 1, NaCl 140; pH = 7.35. Seal resistances were 3-6 GΩ. injected hyperpolarising currents were were -0.86±0.13 pA/pF for early hiPSC-CM and -1.05±0.16 pA/pF for late hiPSC-CM. All recordings were acquired using a HEKA amplifier and HEKA patchmaster software and analysed using Lab Chart 7 (AD instruments, Otago, New Zealand).

Optical APs were measured by loading the cells with 0.1x VoltageFluor2.1Cl (Fluovolt, Thermo Scientific) for 20 minutes in a bath solution containing (in mM): $CaCl_2$ 2, Glucose 10, HEPES 10, KCl 4, $MgCl_2$ 1, NaCl 140, Probenecid 2; pH = 7.35 adjusted with NaOH. Coverslips containing iPSC-CM were transferred to a 37±0.5 °C heated chamber containing bath solution. Cells were electrically stimulated at 0.5 Hz with two parallel platinum electrodes connected to an external stimulator (IonOptix Myopacer cell stimulator). Stimuli were set to 3-5 ms bipolar pulses with voltages ~ 25% above the contraction threshold (10-30 V). APs were recorded from isolated masked cells on the stage of an epifluorescence microscope (λ_{Ex} = 470 nm, λ_{Em} = 535 nm), which was optimised for high-speed signal capture with a photomultiplier as previously described [5]. Three APs from each cell at every

measured frequency were ensemble averaged for offline analysis of AP parameters with Clampfit 10.7 (Molecular Devices).

Western Blot

hiPSC-CMs were dissociated with 0.25% Trypsin/EDTA, snap frozen in liquid nitrogen and stored at -80°C. Cells were lysed on ice with a lysis buffer composed of 20 mM Tris/HCl (pH 7.4), 200 mM NaCl, 1 mM Na_3VO_4, 20 mM NaF, 1% Triton X-100, 1 mM dithiothreitol, and PhosSTOP phosphatase inhibitor (Roche). Cytosol and membranes were separated by centrifugation at 13,000g for 5 min at 4 °C. A bicinchoninic acid assay (Thermo Fisher Scientific) was used to determine protein concentration. Samples were denatured at 37°C for 30 minutes. 40 µg protein was separated by gel electrophoresis (SDS-PAGE) at 25 mV for 60 minutes with a 10% polyacrylamide gel along with a 4.3% stacking gel. Proteins were transferred to a nitrocellulose membrane at 400 mA on ice for 2 hours. Membranes were incubated at 4°C with primary antibodies overnight (**Online Table 1**) diluted in either 2.5% milk or 1% BSA. Blots were imaged using LI-COR Odyssey CLx infrared imaging system and LI-COR C-Digit chemiluminescent scanning system where appropriate.

Flow Cytometry

hiPSC-CMs were dissociated with 0.25% Trypsin/EDTA, then fixed with 4% PFA at RT for 20 minutes, before being permeabilised with 0.1% Triton X-100, and then blocked with 1% BSA in PBS at 4°C for 2 hours. hiPSC-CM were incubated at 4°C with the antibody against alpha-actinin overnight. After washing 3 times, incubation with the secondary antibody proceeded at room temperature using 1% BSA in PBS for 1 hour. The cells were analysed with the LSRII flow cytometer (BD Biosciences).

Unstained and cells were treated as negative controls. A minimum of 10,000 events were analysed per batch.

Immunocytochemical staining

hiPSC-CMs were dissociated with 0.25% Trypsin/EDTA, then fixed with 4% PFA at RT for 20 minutes, before being permeabilised with 0.1% Triton X-100, and then blocked with 1% BSA in PBS at 4°C for 2 hours. hiPSC-CM were incubated at 4°C with antibodies against alpha-actinin and 4′,6-diamidino-2-phenylindole (DAPI) overnight. Cells were imaged using an AxioObserver A1 fluorescence microscope (Zeiss) and Axiovision software 4.70 (Zeiss).

Computational modeling

Simulation tool installation and user guide

❖ Installation of iMATURE

iMATURE is a computational modeling software that employs Myokit, a tool for cardiac cellular electrophysiology simulations developed by Clerx *et al* [1]. Therefore, Myokit needs to be installed according to the installation guide that can be found on www.myokit.org before iMATURE can be installed. These instructions are regularly updated.

Myokit installation: Brief instructions for windows users.

NOTE: these instructions are valid from January 2023 but are subject to change. Please consult www.myokit.org for updated details.

[1] Download and install Miniconda Python 3.8.
- Visit https://docs.conda.io/en/latest/miniconda.html
- Choose the Miniconda3 Windows 64-bit installer link for windows.

[2] Download and install a visual compiler.
- Visit https://visualstudio.microsoft.com/de/downloads/
- Scroll to the end of the page. Select older downloads.
- Select the 2019 option and click download. (Microsoft may ask you to login with a MS account).
- Locate 'Build Tools for Visual Studio 2019 (version 16.9)'. Download and install.
- Open the newly installed Visual Studio Installer.
  - Select the 'Individual components' tab.
  - Use search bar to find and select:
    - MSVC v142 – VS 2019 C++ x64/x86 build tools (latest)
    - C++ Build tools core features
    - Windows 10 SDK (10.0.19041.0)
- Install and download all individual components.

[3] From the windows start menu, open the Anaconda prompt as an administrator and install Myokit by typing: ***pip install myokit[pyqt]***

After Myokit is successfully installed, iMATURE can then be installed. Type in the Anaconda Prompt: ***pip install iMATURE-cardio***

When the installation of iMATURE is finished, iMATURE can be launched by typing in the Anaconda Prompt: ***imature***

The iMATURE user interface, as shown in **Figure 8** of the manuscript, will pop-up and iMATURE is now ready for use.

❖ Running action potential simulations

[1] Click on ‘Cell 1’ or ‘Cell 2’ in the left panel.
  - Insert the preferred maturation stage (in days).
  - If desired, the maximum ion channel conductance of major ionic currents can be altered by adjusting the slider or by entering the desired value in the box to the right of the slider.
  - Press "Run" to start the action potential simulation. This may take a few seconds.

[2] The "Output" panel shows two plots that each depict a particular variable. By default, the top panel shows the membrane potential and the bottom panel shows the calcium transient. The drop-down menus of each plot can be used to select the variable to be shown. The black reference model represents a maturity level of 30 days.

[3] Click on “Options” in the left panel to define the range of the x-axis by entering the desired values.

[4] Click on “Settings” to open a panel that enables changes of stimulus properties, including pacing rate, stimulus duration, stimulus amplitude, hyperpolarising current and number of pre-pacing beats (set to 1000 to reproduce manuscript figures), as well as extracellular ionic concentrations.

[5] To reset all the user-defined values to the default settings, press "Reset Values" followed by "Run" to show the default plots.

❖ Upgrading iMATURE

To upgrade iMATURE into a newer version, open the Anaconda Prompt from the start menu and type: ***pip install --upgrade iMATURE-cardio***

❖ Exporting results

Plots shown in the “Output” panel can be exported as .csv files for further processing. Click on "Export" and press "Export Plot(s)". For each plot, a .csv is generated in which the columns are arranged as followed: Time, Reference, Time, Cell 1, Time, Cell 2.

Supplemental Tables

Online Table 1. Antibodies used for Western blot experiments.

Antigen	Species	Source	Cat. No.	Dilution	Secondary antibody used	Source	Colour	Cat. No.	Dilution
**NCX1**	Rabbit	Swant	p 11-13	1:1000	IRDye® 800CW Donkey anti-Rabbit IgG Secondary Antibody	LI-COR	Green	926-32213	1:10.000
**SERCA2a**	Goat	SantaCruz Biotechnology, Inc	Sc-8095	1:1000	AzureSpectra Fluorescent Secondary Antibody Donkey anti-goat 550	Azure Biosystems	Blue	AC2164	1:2500
**Kir2.1**	Mouse	Hoelzel-biotech	75-210	1:200	IRDye® 680RD Donkey anti-Mouse IgG Secondary Antibody	LI-COR	Greyscale	926-68072	1:10.000
**CSQ2**	Rabbit	Invitrogen	PA1-913	1:1000	IRDye® 800CW Donkey anti-Rabbit IgG Secondary Antibody	LI-COR	Green	926-32213	1:10.000

Online Table 2. Model Parameters

Parameter	Published	Young (30 days)	Old (60 days)	Equation
**Cm**	60	41.88	23.57	Y = 20 + (57.17 - 20)/(1+10^((34.14 - X)*(-0.03764)))
**Caffeine factor**	0	0.001557	0.001557	Y = 0*X + 0.001557
**I_{Na} Block (Conductance)**	0 (100%)	0.721556 (27.8444%) → 45d	0.308444 (69.1556%) → 65d	Y = 0.28 + (0.47)/(1+10^((55 - X)*(-0.1191)))
**I_{Na} shift activation**	0	-5.5 → 45d	-1 → 65d	Y = (-5.5) + (4.5)/(1+10^((55 - X)*(0.1191)))
**$I_{Ca,L}$ Block (Conductance)**	0 (100%)	0.666362 (33.3638%)	-0.548608 (154.8608%)	Y = -0.040499*X + 1.881332
**V Shift of $I_{Ca,L}$ activation**	-20	-25.5	-1.5	Y = 0.80*X - 49.5
**tau of $I_{Ca,L}$ activation**	-7	-5.094197	-9.734387	Y = -0.154673*X - 0.454007
**I_{K1} Block (Conductance)**	0 (100%)	-1.761695 (276.1695%)	-4.901691 (590.1691%)	Y = -11.905593 + (11.905593)/(1+10^((67.682107 - X)*(-0.020176)))
**I_{NaK} conductance factor**	100%	1.518392 (151.8392%)	2.081608 (208.1608%)	Y = 1.5 + 0.6/(1+10^((45-X)*0.1))
**NCX Block (Conductance)**	0 (100%)	0.109269 (89.0731%)	-0.572661 (157.2661%)	Y = -0.022731*X + 0.841199 - 0.05
**SERCA Block (Conductance)**	0 (100%)	0.660598 (33.9402%)	0.801402 (19.8598%)	Y = 0.706 - 0.05 + 0.15/(1+10^((45 - X)*0.1))
**Cytosolic Ca^{2+} buffering (Buf_C)**	0.06	0.224746	0.165556	Y = -0.001973*X + 0.283936
**SR Ca^{2+} buffering (Buf_SR)**	12	12.25319	12.03953	Y = -0.007122*X + 12.466850
**ICaB factor**	1	3.862997	6.684947	Y = 0.094065*X + 0.841047 + 0.2

Supplemental Figures

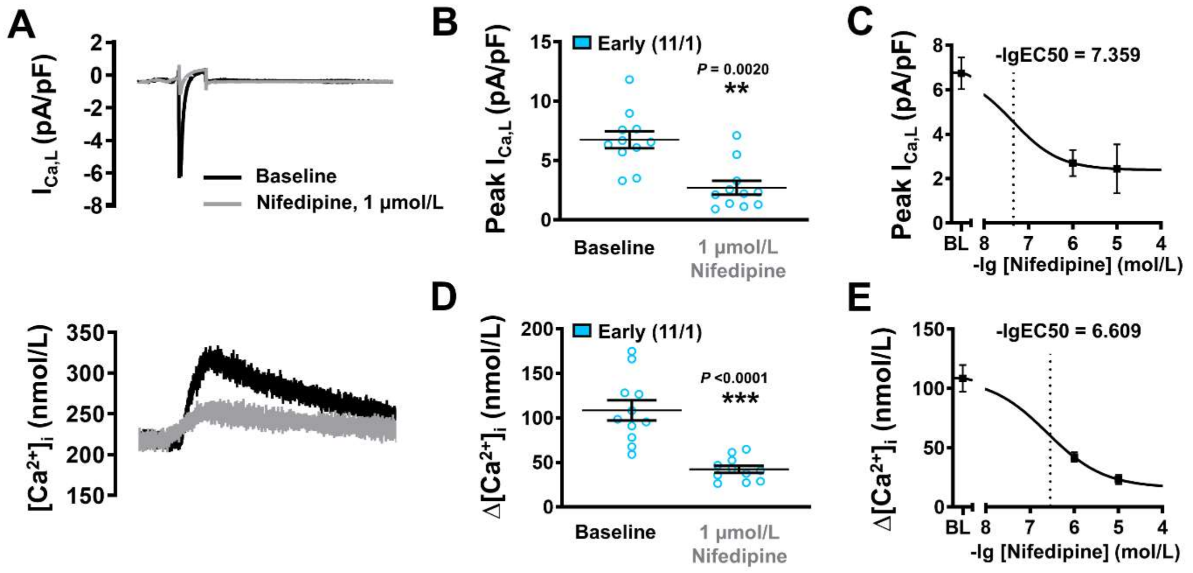

Online Figure S1. Nifedipine-sensitive L-type Ca^{2+} current ($I_{Ca,L}$) and triggered Ca^{2+} transient (CaT) in human induced pluripotent stem cell-derived cardiomyocytes (hiPSC-CM). A, Representative simultaneous recordings of $I_{Ca,L}$ (upper) and triggered CaT (lower) in early hiPSC-CM before and after nifedipine (1 μmol/L) application. **B,** Peak $I_{Ca,L}$ in early hiPSC-CM in the absence or presence of nifedipine. **C,** Concentration response curve for the antagonistic effects of nifedipine on $I_{Ca,L}$ in early hiPSC-CM. **D,** CaT-amplitude in early hiPSC-CM with and without nifedipine application **E,** Concentration-response curve of CaT-amplitude response to nifedipine in early hiPSC-CM. Data are mean±SEM. **P<0.01, ***P<0.001 vs baseline. n/N = number of hiPSC-CM/differentiation.

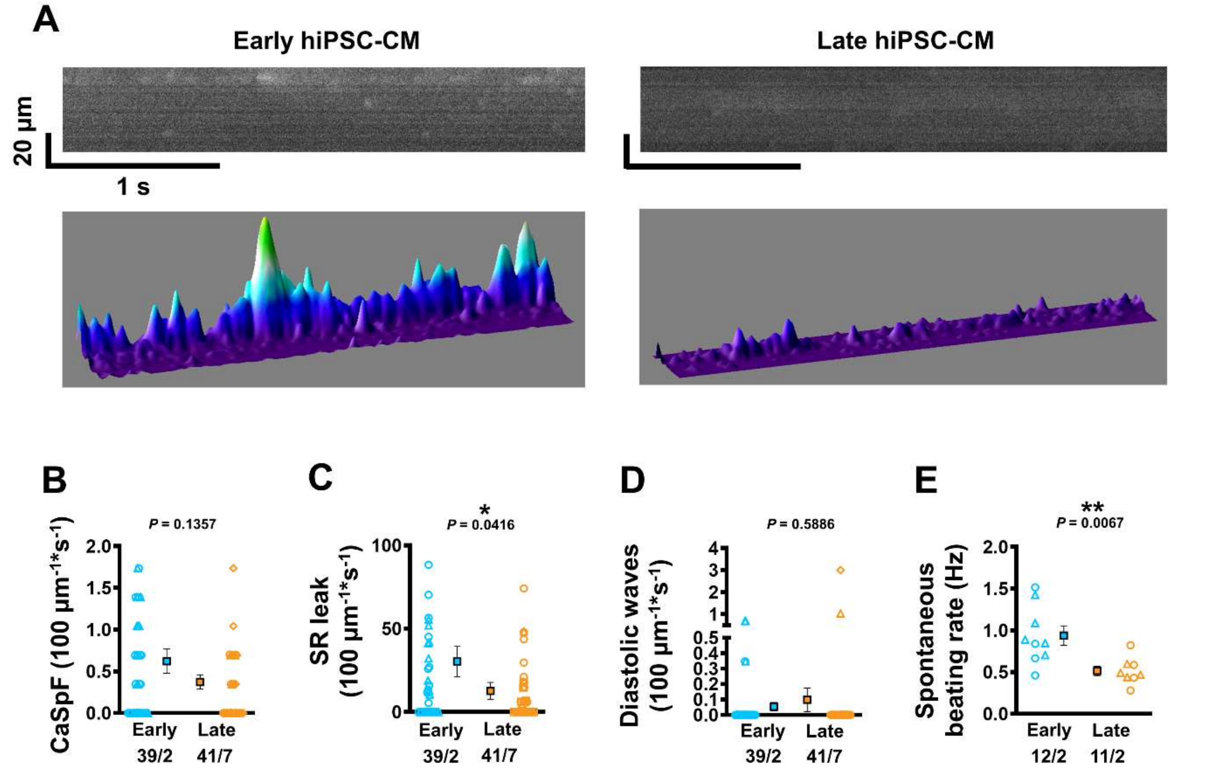

Online Figure S2. Diastolic Ca^{2+} handling in early and late human induced pluripotent stem cell-derived cardiomyocytes (hiPSC-CM). A, Representative line scans of diastolic Ca^{2+} sparks in early (left) and late hiPSC-CM (right) and corresponding 3D surface plots (lower). **B,** Ca^{2+}-spark frequency (CaSpF). **C,** diastolic Ca^{2+} leak from the sarcoplasmic reticulum (SR). **D,** Frequency of detected diastolic Ca^{2+} waves. **E,** Spontaneous rate of cellular contraction. Data are mean±SEM. **P*<0.05 ***P*<0.05 vs early hiPSC-CM culture by Student's t test (**E**) non-parametric Mann-Whitney U test (**C**). Symbols represent separate differentiations. n/N = number of hiPSC-CM/differentiation.

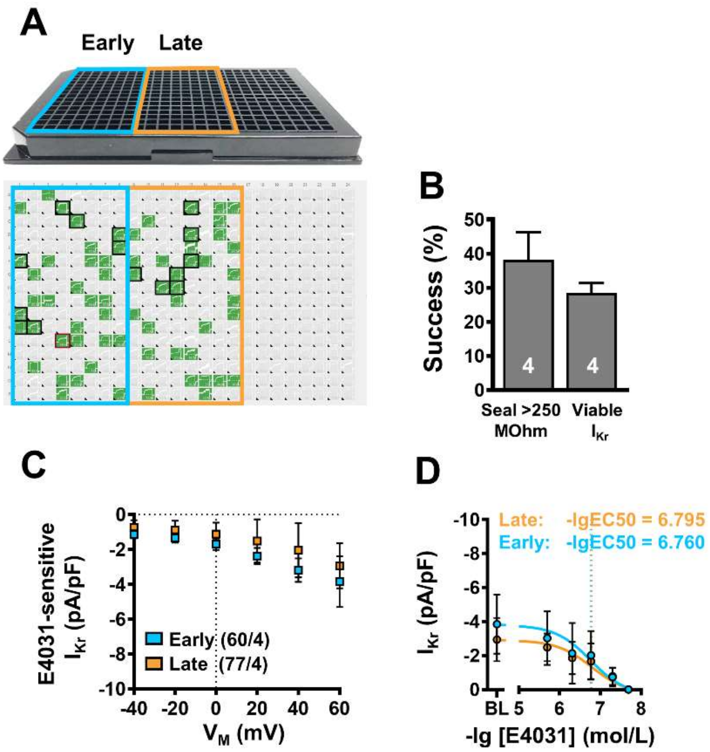

Online Figure S3. Rapid delayed inward rectifier K$^+$ current (I_{Kr}) characteristics and measurement technique. A, Representative 384-well automated patch clamp (APC) chip partially filled with early and late hiPSC-CMs (upper) with screenshot of analysis software showing successful measurements (lower). **B**, APC success metrics. N = 4 chips. **C**, Current-voltage (I-V) curve of I_{Kr} measurements in early and late hiPSC-CM. **D**, Concentration response curves following I_{Kr} block with E4031. Data are mean±SEM. n/N = number of hiPSC-CM/differentiation.

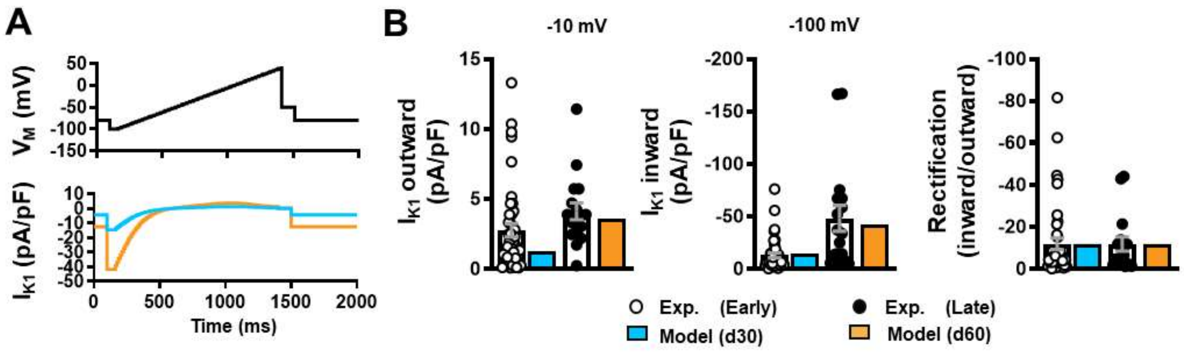

Online Figure S4. Calibration of basal inward-rectifier K⁺ current (I_{K1}) properties incorporating experimentally-observed maturation-dependent changes in early and late human induced pluripotent stem cell-derived cardiomyocyte (hiPSC-CM) models. A, Ramp voltage-clamp protocol (upper) and simulated I_{K1} (lower) in early and late hiPSC-CM. **B**, Outward basal I_{K1} at -10 mV (left), inward basal IK1 at -100 mV (center) and total rectification (right) in experimental and *in silico* early and late hiPSC-CM. The mean ± SEM of experimental data are displayed together with the individual data points. (Experimental data = Exp. and *in silico* model = Model).

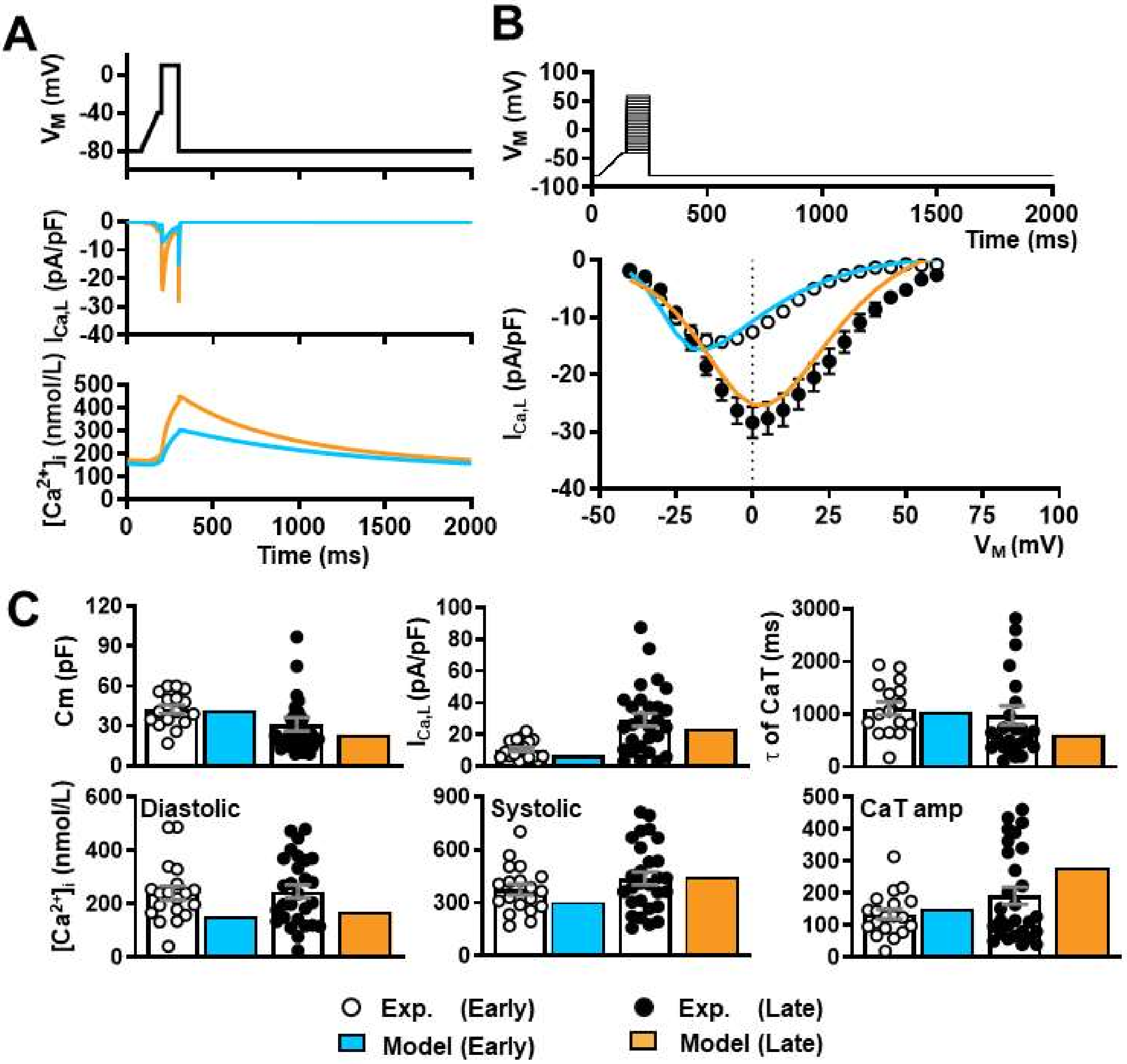

Online Figure S5. Calibration of model parameters of key Ca^{2+}-handling properties in early and late human induced pluripotent stem cell-derived cardiomyocytes (hiPSC-CM). A, Voltage-clamp protocol (0.5 Hz, upper), simulated traces of L-type Ca^{2+} current ($I_{Ca,L}$; middle) and triggered Ca^{2+} transient (CaT; lower) **B,** Voltage-clamp protocol (upper) and $I_{Ca,L}$ current-voltage relationship (I-V) curves in early and late model variants overlaid with experimental data points (lower). **C,** Model and experimental cellular capacitance (Cm; upper left), peak $I_{Ca,L}$ (upper middle), time constant of CaT decay (τ; upper right), diastolic (lower left) and systolic $[Ca^{2+}]_i$ (lower middle) and CaT-amplitude (lower right) in early and late hiPSC-CM. The mean ± SEM of experimental data are displayed together with the individual data points. (Experimental data = Exp. and *in silico* model = Model).

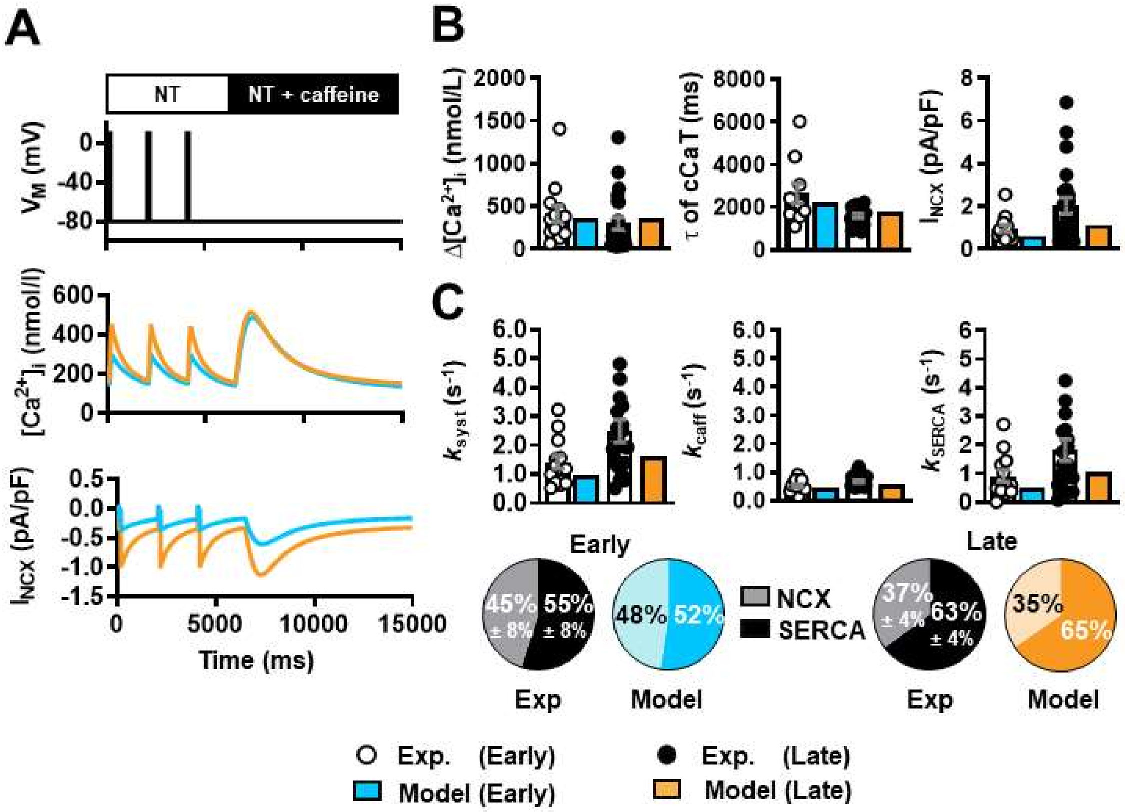

Online Figure S6. Calibration of model Ca^{2+}-handling components incorporating experimentally-observed maturation-dependent changes in sarcoplasmic reticulum (SR) Ca^{2+} content in early and late human induced pluripotent stem cell-derived cardiomyocytes (hiPSC-CM). A, Voltage-clamp and perfusion protocol (three consecutive steady-state beats at 0.5 Hz followed by caffeine infusion to promote SR Ca^{2+} release, upper), simulated Ca^{2+} transients (CaT) and caffeine-induced Ca^{2+} transient (cCaT; middle), corresponding membrane currents (I_{NCX}; lower) in early and late hiPSC-CM. **B,** Model and experimental (Exp.) cCaT amplitude (left), time constant of cCaT decay (τ; middle), peak I_{NCX} (right) in early and late hiPSC-CM. **C,** Rate constants of Ca^{2+} transport k_{syst} (upper left), k_{caff} (upper middle) and k_{SERCA} (upper right; calculated as the difference between k_{syst} and k_{caff}) and relative contribution of NCX and SERCA in experimental and *in silico* early and late hiPSC-CM. The mean ± SEM of experimental data are displayed together with the individual data points. (Experimental data = Exp. and *in silico* model = Model).

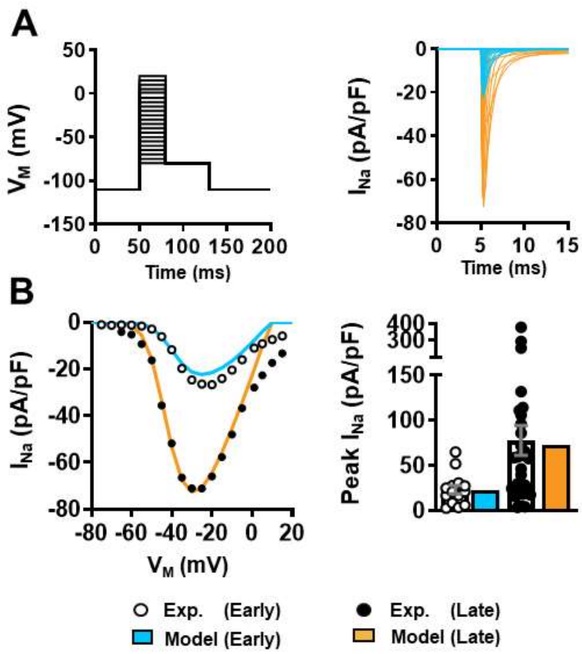

Online Figure S7. Calibration of fast Na⁺ current (I_{Na}) properties incorporating experimentally-observed maturation-dependent changes in early and late human induced pluripotent stem cell-derived cardiomyocyte (hiPSC-CM) models. A, Voltage-clamp protocol (left) and simulated I_{Na} traces (right) in early and late hiPSC-CM. **B,** Simulated current-voltage relationship (I-V) in early and late model variants (lines) overlaid with experimental data points (left); model and experimental peak I_{Na} in early and late hiPSC-CM (right). The mean ± SEM of experimental data are displayed together with the individual data points. (Experimental data = Exp. and *in silico* model = Model).

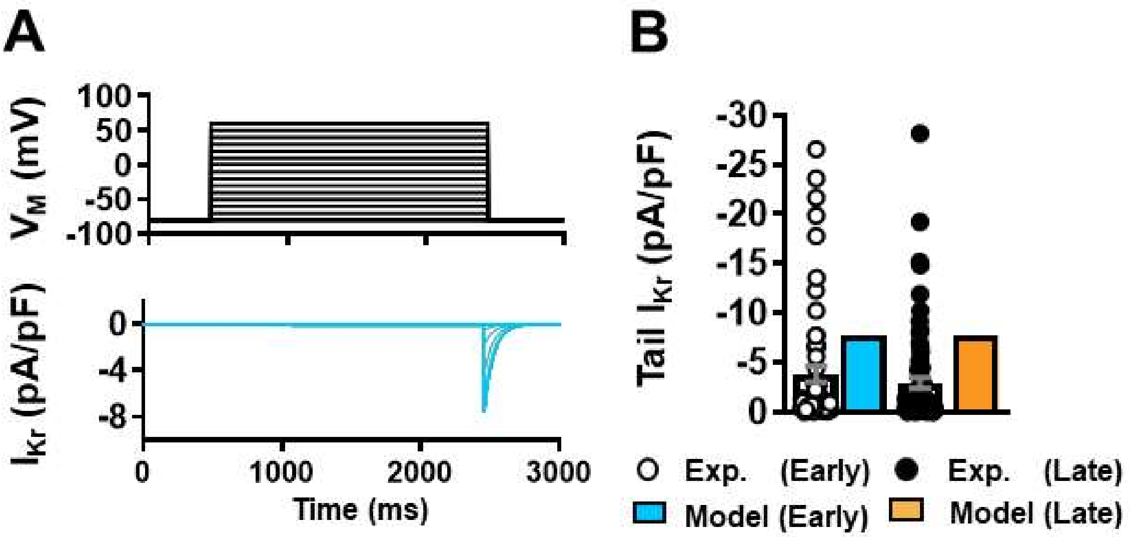

Online Figure S8. Calibration of the rapid component of the delayed rectifier K+ current (I_{Kr}) properties incorporating experimentally-observed maturation-dependent changes in early and late human induced pluripotent stem cell-derived cardiomyocyte (hiPSC-CM). A, Voltage-clamp protocol (upper) and simulated tail I_{Kr} (lower) in early and late hiPSC-CM. Model and experimental tail I_{Kr}. The mean ± SEM of experimental data are displayed together with the individual data points. (Experimental data = Exp. and *in silico* model = Model).

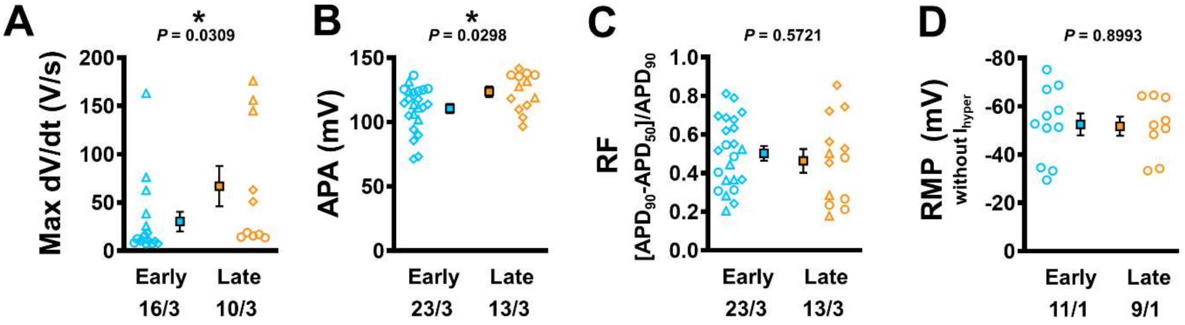

Online Figure S9. Experimental action potential (AP) characteristics in isolated early and late human induced pluripotent stem cell-derived cardiomyocytes (hiPSC-CM). A, Maximum AP upstroke velocity (Max. dV/dt). **B,** AP amplitude (APA). **C,** Repolarisation fraction (RF), a normalised index of repolarisation profile as described by Du *et al* [2]. **D,** Resting membrane potential (RMP) in a separate cellular cohort in the absence of injected current (I_{stim}). Data are mean±SEM. *P<0.05 vs early hiPSC-CM culture. Symbols represent separate differentiations. n/N = number of hiPSC-CM/differentiation.

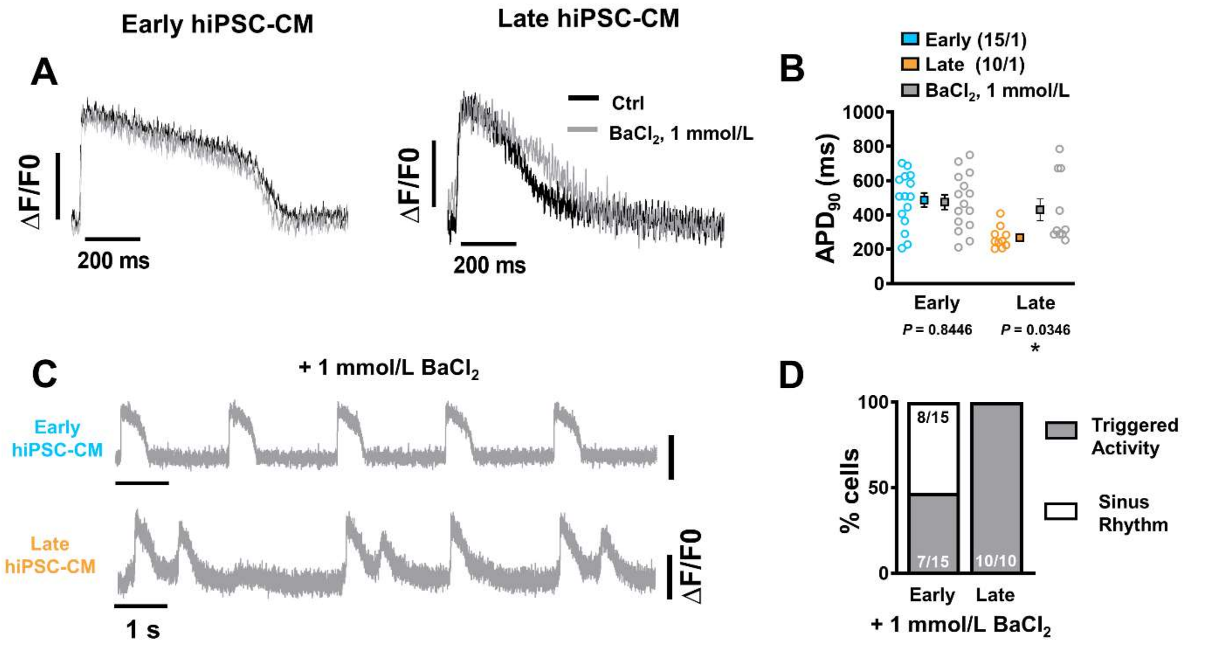

Online Figure S10. Experimental optical action potential (AP) measurement with blocade of I_{K1} with 1 mmol/L $BaCl_2$ in isolated early and late human induced pluripotent stem cell-derived cardiomyocytes (hiPSC-CM). A, Representative recordings of AP in early hiPSC-CM (left) and late hiPSC-CM (right) before and after $BaCl_2$ application. **B,** AP duration at 90% repolarisation (APD_{90}). **C,** Representative traces of membrane potential in early (upper) and late (lower) hiPSC-CM undergoing field stimulation at 0.5 Hz during I_{K1} blockade with $BaCl_2$. **D,** Quantification of arrythmogenic activity in hiPSC-CM during I_{K1} blockade with $BaCl_2$. Data are mean±SEM. **P*<0.05 vs early hiPSC-CM culture. n/N = number of hiPSC-CM/differentiation.

References

1. Clerx M, Collins P, de Lange E, Volders PGA (2016) Myokit: A simple interface to cardiac cellular electrophysiology. Prog Biophys Mol Biol 120:100–114. doi: https://doi.org/10.1016/j.pbiomolbio.2015.12.008
2. Du DTM, Hellen N, Kane C, Terracciano CMN (2015) Action potential morphology of human induced pluripotent stem cell-derived cardiomyocytes does not predict cardiac chamber specificity and is dependent on cell density. Biophys J 108:1–4. doi: 10.1016/j.bpj.2014.11.008
3. Huang M, Liao Z, Li X, Yang Z, Fan X, Li Y, Zhao Z, Lang S, Cyganek L, Zhou X, Akin I, Borggrefe M, El-Battrawy I (2021) Effects of Antiarrhythmic Drugs on hERG Gating in Human-Induced Pluripotent Stem Cell-Derived Cardiomyocytes From a Patient With Short QT Syndrome Type 1. Front Pharmacol 12:675003. doi: 10.3389/fphar.2021.675003
4. Poulet C, Wettwer E, Grunnet M, Jespersen T, Fabritz L, Matschke K, Knaut M, Ravens U (2015) Late Sodium Current in Human Atrial Cardiomyocytes from Patients in Sinus Rhythm and Atrial Fibrillation. PLoS One 10:e0131432. doi: 10.1371/journal.pone.0131432
5. Seibertz F, Reynolds M, Voigt N (2020) Single-cell optical action potential measurement in human induced pluripotent stem cell-derived cardiomyocytes. J Vis Exp 2020:e61890. doi: 10.3791/61890
6. Sossalla S, Kallmeyer B, Wagner S, Mazur M, Maurer U, Toischer K, Schmitto JD, Seipelt R, Schöndube FA, Hasenfuss G, Belardinelli L, Maier LS (2010) Altered Na^+ currents in atrial fibrillation effects of ranolazine on arrhythmias and contractility in human atrial myocardium. J Am Coll Cardiol 55:2330–2342. doi: 10.1016/j.jacc.2009.12.055
